# Revealing solvent-dependent folding behavior of mycolic acids from *Mycobacterium tuberculosis* by advanced simulation analysis

**DOI:** 10.1007/s00894-019-3943-5

**Published:** 2019-02-14

**Authors:** Wilma Groenewald, Ricardo A. Parra-Cruz, Christof M. Jäger, Anna K. Croft

**Affiliations:** 1 School of Chemistry, Bangor University, Bangor, Gwynedd LL57 2UW UK; 2 Department of Chemical and Environmental Engineering, University of Nottingham, University Park, Nottingham, NG7 2RD UK

**Keywords:** Mycolic acids, Folding, Molecular dynamics, Free energy landscapes, Conformational analysis

## Abstract

*Mycobacterium tuberculosis* remains a persistent pathogen, partly due to its lipid rich cell wall, of which mycolic acids (MAs) are a major component. The fluidity and conformational flexibilities of different MAs in the bacterial cell wall significantly influence its properties, function, and observed pathogenicity; thus, a proper conformational description of different MAs in different environments (e.g., in vacuum, in solution, in monolayers) can inform about their potential role in the complex setup of the bacterial cell wall. Previously, we have shown that molecular dynamics (MD) simulations of MA folding in vacuo can be used to characterize MA conformers in seven groupings relating to bending at the functional groups (W, U and Z-conformations). Providing a new OPLS-based forcefield parameterization for the critical cyclopropyl group of MAs and extensive simulations in explicit solvents (TIP4P water, hexane), we now present a more complete picture of MA folding properties together with improved simulation analysis techniques. We show that the ‘WUZ’ distance-based analysis can be used to pinpoint conformers with hairpin bends at the functional groups, with these conformers constituting only a fraction of accessible conformations. Applying principle component analysis (PCA) and refinement using free energy landscapes (FELs), we are able to discriminate a complete and unique set of conformational preferences for representative alpha-, methoxy- and keto-MAs, with overall preference for folded conformations. A control backbone-MA without any mero-chain functional groups showed significantly less folding in the mero-chain, confirming the role of functionalization in directing folding. Keto-MA showed the highest percentage of WUZ-type conformations and, in particular, a tendency to fold at its alpha-methyl trans-cyclopropane group, in agreement with results from Villeneuve et al. MAs demonstrate similar folding in vacuum and water, with a majority of folded conformations around the W-conformation, although the molecules are more flexible in vacuum than in water. Exchange between conformations, with a disperse distribution that includes unfolded conformers, is common in hexane for all MAs, although with more organization for Keto-MA. Globular, folded conformations are newly defined and may be specifically relevant in biofilms.

**Electronic supplementary material:**

The online version of this article (10.1007/s00894-019-3943-5) contains supplementary material, which is available to authorized users.

**Graphical abstract:**
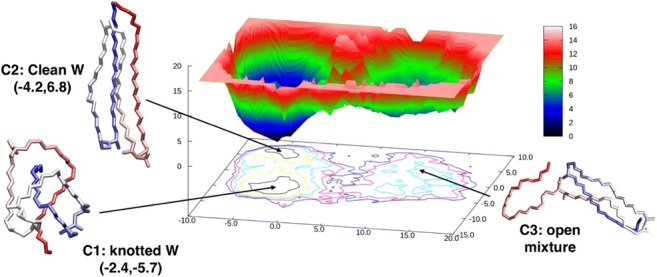
Through advanced simulation analysis, including principle component analysis and free energy landscapes, we reveal detailed physical insights into the solvent-dependant folding behavior of mycolic acids from *M. tb*.

## Introduction

In 2016, an estimated 1.3 million people died from tuberculosis (TB), amounting to more than 3500 deaths per day [1]. This is shocking, in view of the fact that TB is largely a curable disease, although treatments are prolonged and require multiple drugs. The organism causing TB in humans, *Mycobacterium tuberculosis*, is particularly resilient, in part due to a lipid-rich cell wall. Mycolic acids (MAs) are major components of the mycobacterial cell wall [2, 3].

**Fig. 1:**
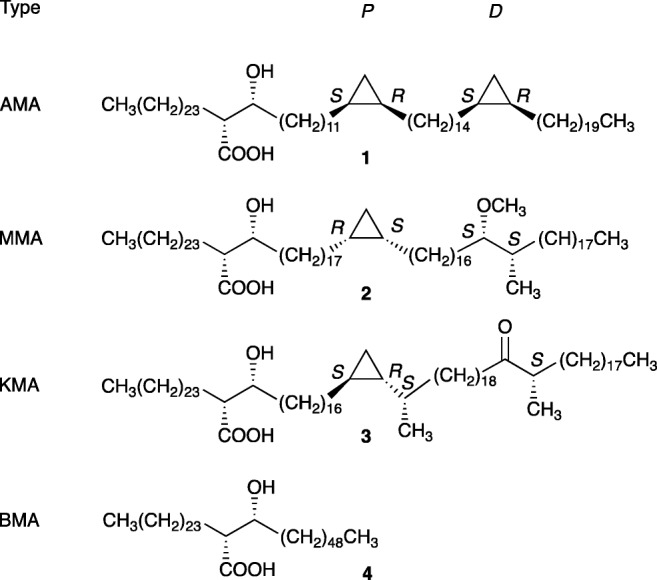
Chemical structures **1**–**4** of mycolic acids (MAs) modelled in this study, representing main components of MAs from *M.ycobacterium tb* [14]. *P* and *D* represent the proximal and distal functional groups, respectively. The backbone MA (BMA) serves as a control and does not have any mero-chain functional groups

MAs are 2-alkyl-3-hydroxy fatty acids with total chain lengths in the vicinity of 60–90 carbons. They mostly occur covalently bound to arabinogalactan, but also exist as trehalose mono- and dimycolates [3–5]. In *M. tb* there are three main classes of MAs that vary at the proximal (*P*) and distal (*D*) functional groups in the long mero-chain, and with the chain lengths between the groups, as shown in Fig. 1. Alpha-MA (AMA) has cis-cyclopropane groups at both *P* and *D*. Oxygenated MAs have a methoxy or keto group with adjacent methyl group at the distal position *D*. The oxygenated MAs exist with either a cis- or trans-methyl cyclopropane group at *P*. In *M. tb* methoxy-MA (MMA) occurs mostly with *cis*-cyclopropane, whereas keto-MAs (KMA) generally have trans-methyl cyclopropane groups [6]. The absolute stereochemistries of the functional groups have been proposed as shown in Fig. 1, obtained through comparison of natural compounds with synthetic compounds [4, 7–13].

There is now significant evidence that particular MAs and specific MA functionalities have a measurable impact on cell wall permeability, growth, virulence, bacterial proliferation, host immune response, and impact on infected cells [15–30] (reviewed also in [4]). Here, two key features play a significant role; oxygenation of the MA [15–23], and cyclopropanation [24–27], including the specific stereochemistry of the cyclopropane ring [22–25]. Confirmation of the role of individual MA structures and benchmarked mixtures has recently been achieved through the use of synthetic derivatives [20–23, 31–33], and strongly implies that the underlying MA structure (individually or as sugar-ester derivatives), which steers the physical properties and conformational behavior of the molecule, can offer a window to rationalize their biological role.

MAs are central to the host immune response against the organism and have been shown to be good antigens for use in serodiagnosis of TB [30, 31, 33–36]. However, fundamental details such as how MAs are arranged in the cell wall, and how they interact with immune components, are yet to be determined. Cryo-electron microscopy results suggest that the outer bilayer of the mycobacterial cell wall is 7–8 nm wide and consists of a symmetrical bilayer structure [37–40]. This observation implies that longer MAs need to fold to fit into this space. Zuber et al. [37] have suggested that MAs fold at each of their functional groups, forming a W-conformation, and intercalate with lipids in the opposite leaflet in a zipper model. Interactions with components of the host immune system, such as antibody binding, are likely to involve a macrostructure consisting of numerous MAs. Therefore, knowledge regarding the preferred conformations of single MAs will provide building blocks for these larger structures and shed light on these areas.

Numerous studies on MA monolayers, which serve as a close approximation of MAs in the cell wall, have been performed experimentally [41–49]. These studies have shown that MA conformations change as the molecules are packed closer together at higher lateral pressures. MAs are suggested to have folded conformations with up to four chains arranged in parallel, as in the W-conformation, occupying a large surface area at low lateral pressures [41–45]. The long mero-chain in AMA and MMA extends completely as the surface area is decreased. In contrast, KMA forms a very rigid monolayer and the molecules stay in a folded W-conformation, even when high lateral pressure is applied.

Villeneuve and co-workers have performed short simulations on various MAs in a W-conformation, studying their preference for staying in the folded conformation or unfolding [42–44]. Their findings supported monolayer results; AMA and MMA tended to unfold, while KMA mostly remained folded [42]. They have found that MAs with alkyl chains of similar length between the functional groups fold into more tightly packed W-conformations and unfold more slowly than MAs in which the alkyl chain lengths differ. The presence of a double bond favored an energetically more stable W-conformation, as compared to cis-cyclopropane [43]. An α-methyl trans-cyclopropane group within the molecule was also found to promote folding of an alkyl chain as compared to the cis-isomer [45].

**Fig. 2:**
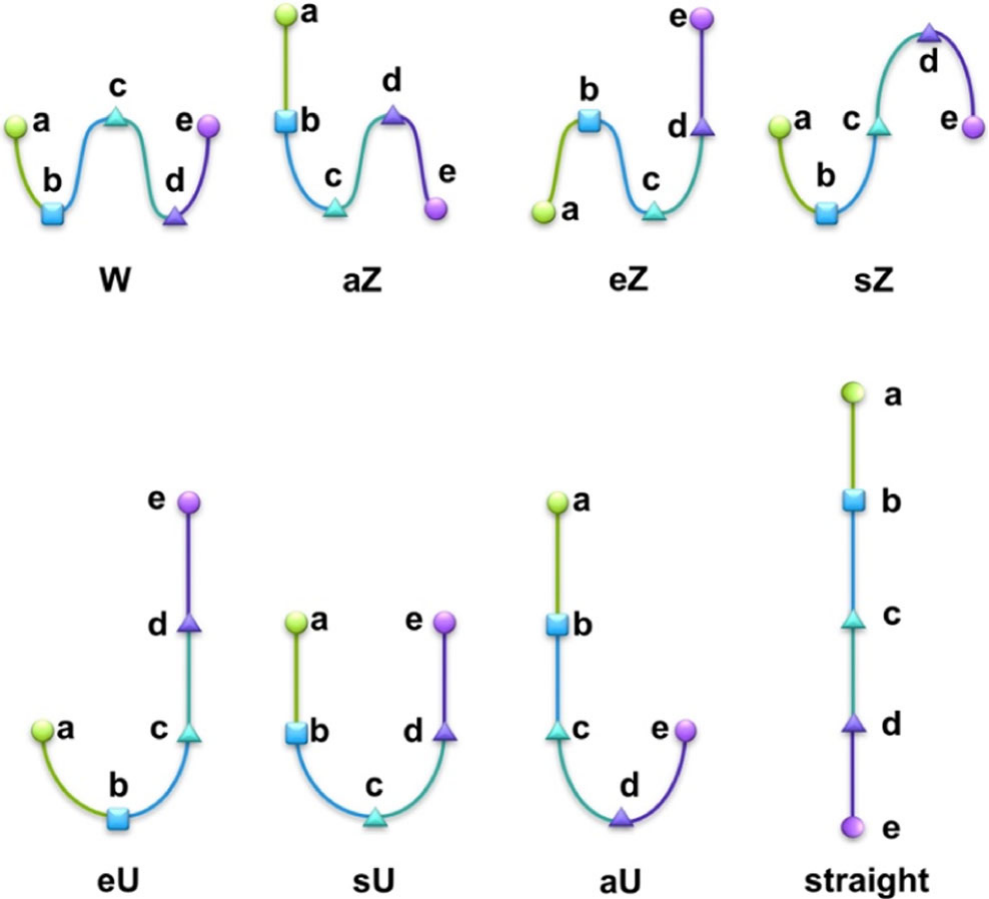
A schematic representation of the seven possible WUZ-conformations and straight conformation. The acid head group, *b*, is indicated by a *square*, and the proximal (P) and distal (D) functional groups, *c* and *d*, by *triangles* [e.g., both cyclopropane for alpha-MA (AMA)]

We have previously shown through unconstrained simulation in vacuo that MAs spontaneously fold into reproducible conformational groupings [50]. Clear differences in conformational preferences between MA classes highlight that the underlying chemical composition steers MA conformation, with KMA showing very different trends to AMA and MMA, consistent with biological and monolayer observations. MAs were categorized through seven possible ‘WUZ’-folds, defined as folding at some or all of their functional groups, with two, three or four alkyl chains in parallel (Fig. 2). Significantly, MAs folded into the WUZ-conformations spontaneously, without any solvent or neighboring MA molecules to aid in the folding, with implied stabilization from van der Waals interaction between the parallel alkyl chains. However, conformations of MAs defined by WUZ only accounted for a small percentage of MA conformations. In this work, we contribute new OPLS parameters to describe more accurately the cyclopropyl group in mycolic acid simulations. We also examine both the impact of explicit solvent on folding patterns and extend our folding descriptions to other MA conformations that have not been described to date, thereby providing a more complete picture of both the intrinsic and external factors that affect MA conformational preferences.

## Methods

### Molecules

Four different MAs **1**–**4** (Fig. 1) were modelled, with the backbone MA (BMA) serving as a control MA containing no functional groups in the mero-chain. Molecules were built using WebMO [51] and Aten [52] graphical interfaces, and were numbered serially from the alpha chain through to the mero-chain. The OPLS all atom (AA) forcefield (with additional parameters for cyclopropane described below) was applied in Aten, and the residue topology parameter file exported. Subsequently, topologies were created using the Gromacs simulation package [53–56]. Each molecule was placed in the centre of a 10 × 10 × 10 nm box. The structures were minimized with a steepest descent algorithm, a maximum step-size of 0.01 nm, a maximum number of 200,000 steps, and a tolerance of 10 kJmol^−1^ nm^−1^.

### Cyclopropane parameters for OPLS all-atom forcefield

For a correct representation of the conformational behavior of the cyclopropane entities of the MAs simulated, the OPLSAA force field parameters had to be improved. Six bonded parameters for cyclopropane, listed in Tables 1 and 2, were obtained using hybrid ensembles for force matching, as detailed elsewhere [57]. Low energy conformers for cyclopropane are approximated well, compared to the reference quantum mechanics data (see Figs. S1–S6). However, there is still room for improving the overall fit of the force field data to the quantum mechanics data. Although the parameterization is not the focus of this work, the addition of the angle parameter, which shows a good fit to reference quantum mechanics data, provides an improvement to describing the cyclopropane unit with this force field.

**Table 1:** Angle (degrees) and force constant (kJmol^−1^ rad^−2^) for cyclopropane angle parameter

Angle	th0	cth
CT–CT–CY	115.090	612.010

**Table 2:** Torsional Fourier coefficients (kJmol^−1^) obtained for cyclopropane

Dihedral	V_1_	V_2_	V_3_	V_4_
CT–CT–CT–CY	1.00000	−22.00200	24.05800	−20.89300
CT–CT–CY–CY	1.00000	14.17700	−20.14000	−9.78690
CY–CT–CT–HC	1.00000	−32.91200	25.29700	2.47730
CT–CT–CY–HC	1.00000	1.43810	21.88000	12.80200
HC–CT–CY–HC	1.00000	9.99550	11.63500	−14.48900

### Simulation details

For all simulations, unless stated otherwise, a timestep of 1 fs was used and the neighbor list was updated every 10 fs. Van der Waals interactions were modelled by using a shift function between 0.8 and 0.9 nm, and electrostatic interactions were modelled by using PME. V-rescale temperature coupling was used at 300 K with a time constant of 0.5 ps and no constraints were applied. The equation of motion was integrated using a leap-frog algorithm. All simulations were performed with Gromacs version 4.5.4 [53]. Vacuum simulations were performed with a NVT ensemble.

Simulations in water were performed by filling the simulation box with TIP4P water. The number of water molecules for the different mycolic acid boxes ranged from 32,951 to 32,972. Equilibration was performed with position restraints by applying a force of 1000 kJ mol^−1^ nm^−2^ in the *x*, *y* and *z*-directions on all carbon and oxygen atoms of the MA. The system was equilibrated by performing 100 ps NVT, followed by 50 ps NPT molecular dynamics (MD) using Berendsen pressure coupling to scale the box in an efficient way at the beginning of the simulations, and lastly 100 ps NPT using Parinello-Rahman pressure coupling to yield the correct ensemble. In both NPT-ensembles, isotropic pressure coupling at a pressure of 1 bar was used with a 1 ps time constant. Production simulations for MAs in water also used the Parinello-Rahman setup and no constraints were applied.

In order to use hexane as a solvent, a hexane solvent box was built and equilibrated before addition to MA simulation boxes. This was done by building a single hexane and obtaining its topology parameter file with Aten. Then a 3.6 × 3.6 × 3.6 nm box was filled with 216 hexane molecules, minimized and equilibrated at 300 K using a NVT ensemble for 5 ns. The energy plots for this equilibration are shown in Fig. S7. The density of the hexane box was 584.201 kg.m^−3^, which is approximately 11% lower than the experimental density for hexane. Simulation boxes containing MAs were filled with hexane using the equilibrated hexane box. The number of hexane molecules ranged from 4159 to 4173 for the different MAs. Equilibration was performed with position restraints by applying a force of 1000 kJ mol^−1^ nm^−2^ in the *x*, *y* and *z*-directions on all carbon and oxygen atoms. The system was equilibrated by firstly increasing the timestep from 10^−7^ ps to 10^−3^ ps by running respective simulations of 100,000 steps at 0 K with a timestep of 10^−7^ ps, followed by a 10^−6^ ps timestep, a 10^−5^ ps timestep, a 10^−4^ ps timestep, and, finally, a 10^−3^ ps timestep with Berendsen pressure coupling using a time constant of 1 ps. Secondly, using a 10^−3^ ps timestep and Berendsen pressure coupling as above, the temperature was increased in 50 K increments from 0 K to 250 K with consecutive 100 ps simulations and a 1 ns simulation at 300 K. Production simulations with hexane as solvent were run using Parinello-Rahman pressure coupling at a pressure of 1 bar and a time constant of 1 ps, a timestep of 2 fs and with all bonds constrained.

For each MA in each of the three different environments, 20 simulations were run for 10 ns each. Starting conformations for the 20 replicate production simulations for each system were varied in order to increase sampling of the potential energy surface. Taking the starting frames 250 ps apart from an initial 5 ns simulation in vacuum and water systems, and 240 ps apart for those in hexane ensured a variety of starting conformations. For each simulation, 1001 frames were written (consisting of one frame every 10 ps, as well as the starting frame at 0 ps) and used in subsequent analysis.

### Conformational analysis

The initial conformational analysis was based on the definition for WUZ conformations defined previously [50]. The backbone carbon chain consists of all the consecutively linked carbons along the length of the MA chain and excludes any non-carbon atoms, the carbon of the acid group, and the CH_2_ carbons of cyclopropyl groups and adjacent methyl groups. A straight reference MA of each type had all its backbone carbon atom dihedrals set to 180°.

**Fig. 3:**
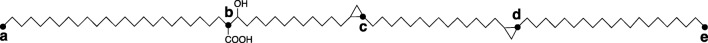
Points *a*–*e* that were used in analysis are indicated on AMA with *dots* and the relevant *letters*

Five points (a–e, Fig. 3) were used to analyze the fold of the molecule [42] with (a) the last carbon in the 2-alkyl chain, (b) the carbon on which the carboxyl group is attached, (c) the distal carbon of the cyclopropane ring, (d) the carbon to which the keto- or methoxy group is attached and in the case of AMA the distal carbon of the cyclopropane ring, and (e) the end carbon of the meromycolate chain.

MA key distances were used to define the seven possible W, U and Z-conformations that describe conformers with hairpin bends at the functional groups [50]. These conformations are shown schematically in Fig. 2 and the criteria are outlined in Table 3. Prefixes “a” and “e” describe conformations in which the a- and e-terminating chains are extended, while “s” refers to symmetry. For each MD run, a python script was used to label all 400 frames as one of these seven folds if they met the criteria for that fold.

**Table 3:** The definitions for the intramolecular distance boundaries for the seven possible WUZ-conformations. For each fold, the chain extensions were defined as ab and de >50% of maximum extension, and bc and cd >70% of the straight-chain chain-length maximum

Fold	Distance between points (nm)			
	ac	ae	ce	bd
W	< 2.0	< 2.5	< 2.0	< 1.0
aZ	> 2.0	> 2.5	< 2.0	< 1.0
eZ	< 2.0	> 2.5	> 2.0	< 1.0
sZ	< 2.0	< 2.5	< 2.0	> 1.8
eU	< 2.0	> 2.5	> 2.0	> 1.8
sU	> 2.5	< 2.5	> 2.5	< 1.0
aU	> 2.0	> 2.5	< 1.8	> 1.8

### Principle component analysis and free energy landscapes

Principal component plots from the either complete data (Fig. S12) or the last 4 ns of simulation data (Fig. S13), were produced for each MA and indicate the degree of variation in folding based on distance criteria relating to backbone carbon atoms. The unfunctionalized BMA was plotted onto the map for AMA for direct comparison, since they share an identical backbone. The WUZ structures are indicated on the plot and consist of minimized average structures, manually identified from the simulations to represent the most idealized conformers. For MMA and KMA sZ was adapted from the average W-conformation by adjusting the angles around the cyclopropane group, c, to 180° due to the small percentage of sZ conformers available and their shape not conforming well to the idealized sZ shape. PCA trajectories were plotted against time to confirm differences in sampling based on simulation time.

Further analysis was achieved through free energy landscapes (FELs) that afford a more nuanced interpretation of conformer distribution and indicate the most stable conformers [58]. FEL were calculated using joint probability distribution from the essential plane constructed from the first two eigenvectors, PC1 and PC2. Conformations were sampled during the simulation and projected on this two-dimensional (2D) plane, and the free energy for each grid cell was calculated using the expression:

$$ \varDelta \mathrm{G}=-{K}_BT\; In\frac{p\left({N}_i\right)}{p\left({N}_{ref}\right)} $$

where *p*(*N*_i_) is an approximation of the probability density function gained from a histogram of molecular dynamics data, and *p*(*N*_ref_) is the maximum of the probability density function; *K*_B_ is the Boltzmann constant, and T is the temperature of the simulation.

Subsequently, minima positions have been selected on the FEL manually. A customized script was used to search for the local minima around the hand-selected minima positions and find all PC combinations within a selected radius around these minima that fulfil the criteria of lying within a defined free energy threshold around the corresponding minimum. All structures fulfilling these criteria were then grouped into clusters and further analyzed. FELs for AMA, MMA and KMA, in each solvent are presented in the Supporting Information, including clustering analyses and a comparison of % coverage of WUZ vs FEL-defined clusters of the simulation space.

## Results and discussion

### Equilibration

All simulations reached thermal equilibration early on (energy, temperature and pressure plots in Figs. S8–10), as simulations in water and hexane underwent preceding equilibration steps and there are very few degrees of freedom to equilibrate in the vacuum simulations. Radius of gyration (Fig. S11) was checked as a measure that gives an indication of the shape of the molecule at each time. This showed convergence in the molecular shape after 6 ns for most of the replicate simulations, when simulated in water (consistent with the hydrophobic chains folding to reduce the surface area exposed to water). Convergence is not seen in vacuum nor in hexane, with hexane showing the most variation in structure. To ensure a consistent set of equilibrated structures were considered, MA conformations from the last 4 ns of each simulation were used. The analyses were also compared to the results of the full simulations to account for the broader range of structures accessed during the 6 ns ‘equilibration’ period.

### Defined WUZ MA conformations

**Fig. 4:**
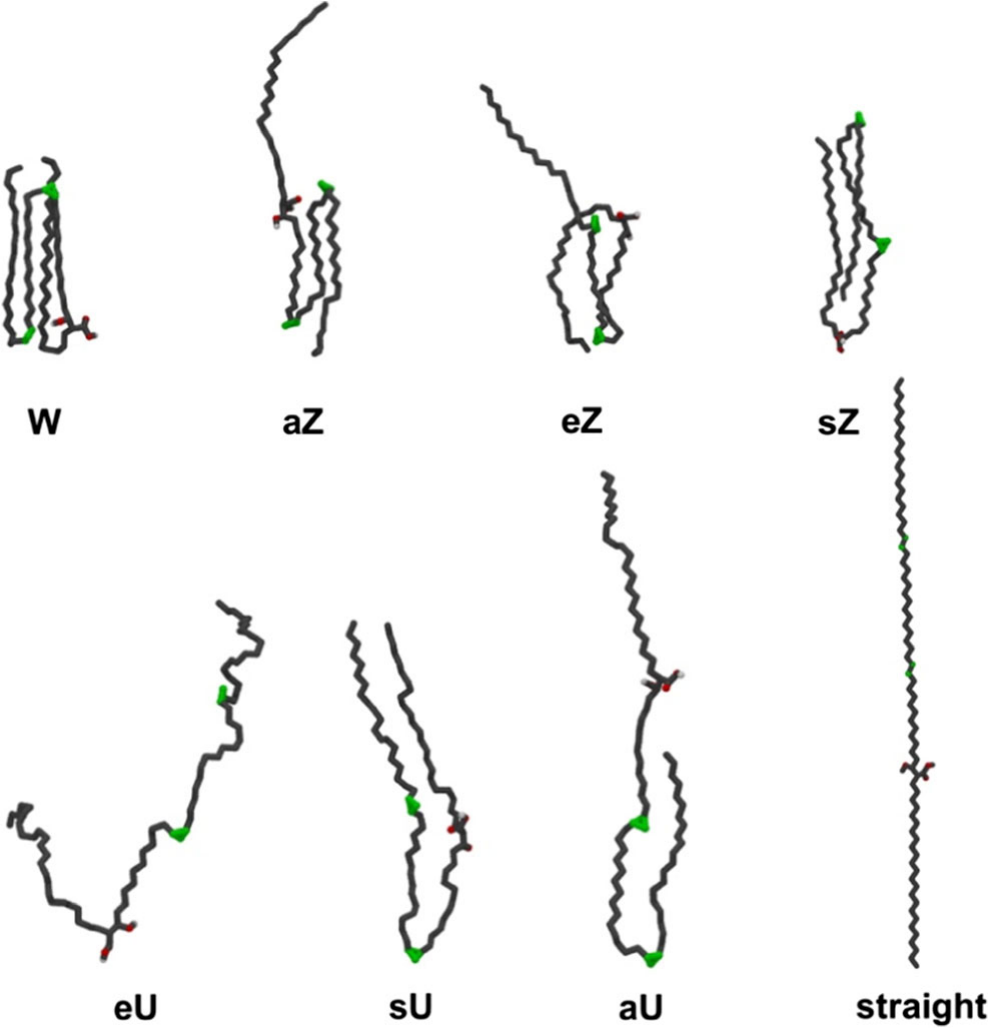
Average structures of the seven possible WUZ-conformations for AMA

Each frame from the simulations was analyzed for the seven possible WUZ-folds according to their chain lengths and intramolecular distances, as defined in Table 3 and shown schematically in Fig. 2. The seven possible WUZ conformations for AMA are shown in Fig. 4. A W-conformation represents bending at each functional group with four parallel alkyl chains. The various Z-conformers fold at two of the functional groups while U-conformers only fold at one functional group.

### Mycolic acid class in relation to WUZ folds

From the WUZ-distributions (Table 4), KMA stands out as having the highest percentage of WUZ-conformations in each of vacuum, water and hexane (48.0%, 27.7% and 29.4%, respectively). This large percentage of WUZ-conformers for KMA clearly distinguishes it from the other MAs, and this very different pattern of folding may correlate with specific biological functions for this MA class [18], as has already been suggested [42, 45]. The difference in the percentage of WUZ-conformations to previous work is presumably due to extended timescales in the current work and an improvement in the description of the cyclopropane group in the forcefield that was used. Both AMA and MMA show fewer WUZ-conformations (11.5%, 15.0% and 10.4% for AMA and 12.8%, 6.5% and 6.9% for MMA in vacuum, water and hexane, respectively) with the backbone control molecule without mero-chain functional groups, BMA the least (3.7%, 1.2% and 5.9% in vacuum, water and hexane, respectively). The reduced number of WUZ-conformations for BMA suggest that the mero-chain functional groups substantially influence how MAs fold, and that the functional groups may steer conformations in unique ways dependent on the chemical structure of each molecule. The different pattern of folding between solvents for AMA vs both MMA and KMA implies a specific solvent effect in the oxygenated MAs, built in water from a lack of disruption of the W-fold for AMA.

**Table 4:** The percentage, calculated as a percentage of the total number of frames for the 20 10 ns simulations of each mycolic acid (MA) of WUZ-conformers obtained for each molecule modelled in vacuum, water and hexane

MA	Solvent	W	aZ	eZ	sZ	eU	sU	aU	Total %
AMA	Vacuum	7.9	1.6	0.2	0.5	0.0	1.0	0.3	11.5
	Water	9.0	5.4	0.0	0.3	0.1	0.0	0.2	15.0
	Hexane	0.0	0.5	0.1	0.3	2.7	1.0	5.8	10.4
MMA	Vacuum	10.3	0.7	0.7	0.0	0.2	0.9	0.0	12.8
	Water	4.4	0.1	1.4	0.0	0.5	0.0	0.0	6.5
	Hexane	0.1	0.5	0.4	0.1	1.8	1.7	2.3	6.9
KMA	Vacuum	39.5	7.3	0.6	0.0	0.0	0.4	0.2	48.0
	Water	19.2	7.7	0.0	0.0	0.0	0.3	0.5	27.7
	Hexane	0.2	7.3	1.1	0.3	0.9	16.4	3.3	29.4
BMA	Vacuum	0.7	0.3	0.2	0.7	0.4	1.0	0.4	3.7
	Water	0.3	0.3	0.1	0.1	0.3	0.0	0.2	1.2
	Hexane	0.0	0.0	0.1	0.1	3.8	0.2	1.7	5.9

### Solvent effect on WUZ folding

In terms of solvent trends, the most compactly folded W-conformation comprises a majority of the identified WUZ conformers simulated in either vacuum or in water. In contrast, mostly more open U-conformations are found in hexane. These results, taken together, emphasize the role of interchain-interactions, where only hexane can behave competitively to break up this structuring. This is further emphasized by the similarity of the WUZ-conformation distributions of BMA. This latter molecule does not have folding directed by functionality, and thus minimal directed chain–chain interactions. For example, in hexane, both AMA and BMA display similarly low levels of WUZ-folding, except for a marginally higher percentage of eU conformers for BMA (3.8%) compared to AMA (2.7%). With folding at the acid head group in the eU conformer, this result suggests that the presence of the remaining functional group, namely the head group, in BMA, facilitates folding at this position. For KMA, however, a significant proportion of sU conformer is present in hexane, and the same level of aZ conformer is retained as in the vacuum and water simulations. This structuring is consistent with an interaction between the meromycolate group and the keto functionality at the distal position, but without further folding to the W-fold as might be driven by stronger inter-chain interactions in water and vacuum. The meromycolate-keto interaction would be hydrogen-bonding in nature, and thus hard to disrupt in hexane. Although MMA, which also has an oxygenated group at the distal functional group, might also be expected to have hydrogen-bonding interactions, the methoxy group is less polar than the keto group and the methyl group may hinder hydrogen bonding in MMA. This suggests additional complexity in determining conformation preference. In addition to potential meromycolate-keto interactions, the sU conformation requires a hairpin fold at the α-methyl trans-cyclopropane group, which has recently been described as facilitating folding in MAs [45]. KMA is the only MA with an α-methyl trans-cyclopropane group modelled here.

In general, the percentages of WUZ-conformations found here are higher than previously found [50], most notably for KMA showing the highest total percentages in all environments simulated. This is attributable largely to the improvements in the force field used, and may also be affected by simulation length and hence sampling of the potential surface. It remains significant that MAs can fold in such structured conformations as are found in ordered compressed monolayers or the cell wall, even when in isolation without any restrictions or lateral packing effects. Nevertheless, WUZ-conformations, comprising hairpin bends at various or all functional groups with straight alkyl chains in parallel and close each other, account for only a fraction of the conformations sampled in simulations of single MAs. WUZ-conformations, in being defined by a restricted number of 2D intramolecular distances, provide a limited description of MA folding. The applied distance cut-offs and the two-dimensionality of the definitions mean that molecules that resemble WUZ-conformations well can be excluded, and conformations that do not resemble the correct three-dimensional (3D) shape are sometimes included in WUZ-defined folds. Hence, to describe the conformational behavior of free MAs in solution more holistically, it is necessary to further develop a well-defined analysis strategy.

### Exploring the wider scope of MA conformations

A more comprehensive picture of the spread of all the conformations was initially obtained by a principle component analysis (PCA) using the carbon backbone of average WUZ-structures combined from extracted frames to map out two principal coordinates for each MA. These principal component plots are shown in Figs. S12 and S13.

The plot for each MA is unique, as the axes are vectors that display the maximum amount of variance for each molecule. At the extremes of the *x*-axis is an extended straight conformation and an sU-like conformation, correlating this axis with the extension of the backbone of the MA. The *y*-axis separates those conformations with the chain terminating with “a” being extended from those terminating with “e” extended. The potential energy surface of each molecule was sampled well when the projection of all conformers from all 20 simulations, sampling a large area, is compared to the projection of single simulation conformers, which sample only a small portion of the conformational space (Fig. S12). This provides confidence in the use of nanosecond timescale and multiple simulations with varied starting conformations in improving the sampling of the conformational space.

A second set of PCA analyses comparing the full sampling trajectories (20 × 10 ns) with the truncated simulations (20 x last 4 ns) shows that, most marked in water, MA conformers converged toward the latter half of the simulation time (consistent with other equilibration measures), indicating different sampling of conformational space between the starting and final conformations (Fig. S14). When the conformers from the last 4 ns of each simulation are projected on the principal component plot (Fig. S13), vacuum and water simulations have conformers grouped more specifically at folded conformations such as W and sZ. More extended conformers are not present in the last 4 ns in vacuum and water. In hexane, the conformer spread remains diffuse with extended conformers even in the last 4 ns. Plots with conformers from the last 4 ns allow the most populated conformations to be more clearly visible.

**Fig. 5:**
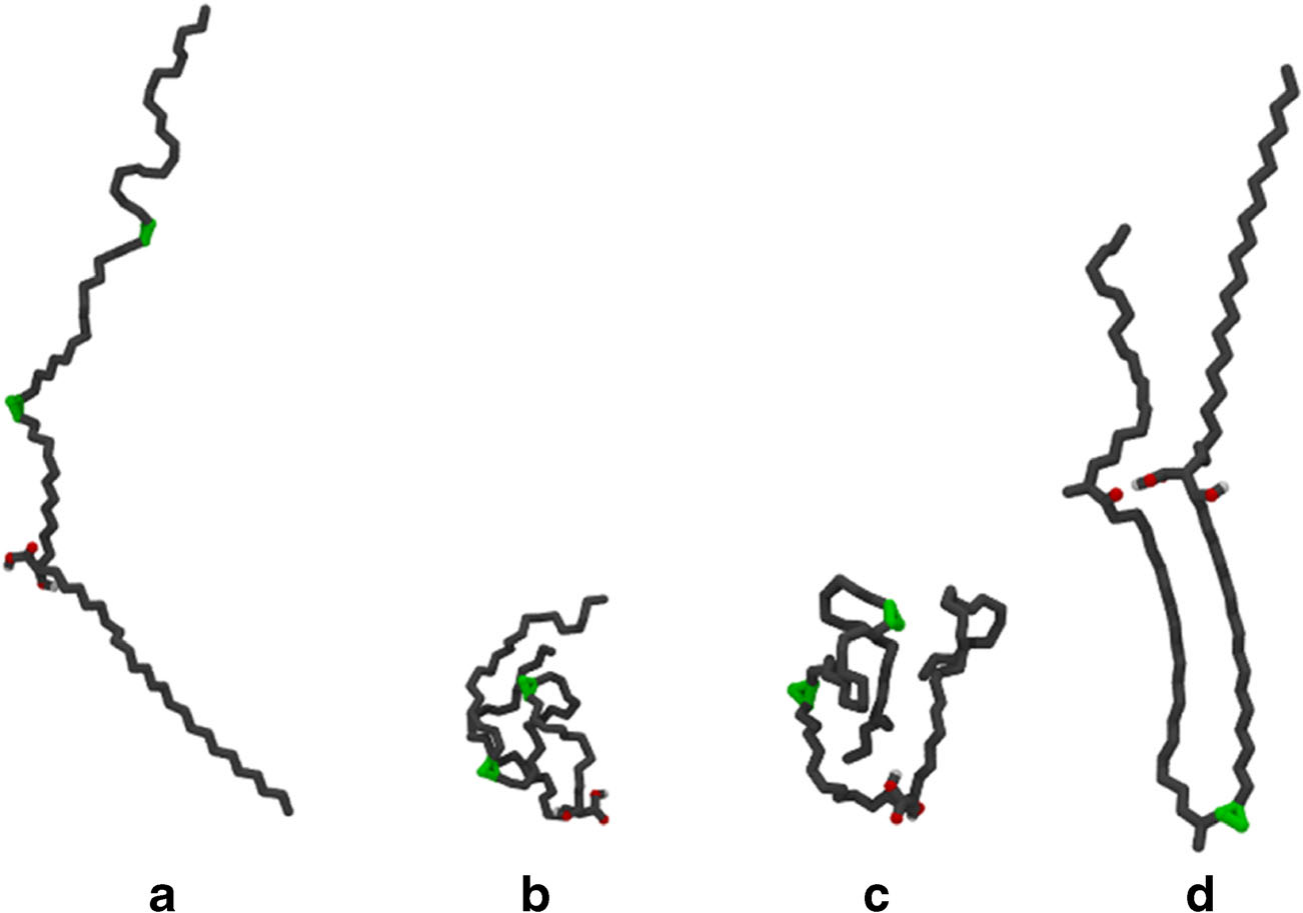
Newly defined conformations for MAs. Structure *A* was obtained from open conformations of AMA in hexane, structures *B* and *C* were obtained from folded conformations of AMA in water, and *D* was obtained from folded conformations of KMA in hexane

WUZ-defined conformations are positioned peripherally to the sampled conformations (Fig. S13). Extraction and averaging of conformers from defined parts of each principal component plot indicated new conformations that differ from the WUZ-defined conformations. The letters A, B, C and G in Fig. 5 represent an example of newly characterized conformers. For each of the MAs, except KMA, a new conformation representative of a large proportion of conformers occurring in hexane was defined. This conformation shown in Fig. 5, A is representative for those indicated by A (AMA), D (MMA) and J (BMA) in Fig. S13. This new conformation is unfolded with a slight bend along the length of the molecule and various kinks in the alkyl chain. In addition, new conformers that are compactly folded, representative of those surrounding the W-conformation (Fig. S13, B and C), are shown in Fig. 5, B and C. These structures are globular in shape with numerous bends, twists and kinks in the alkyl chains. The chains weave between each other in diverse patterns, making these conformations hard to define in two dimensions. Similar globular conformations were found located at E and F (MMA), H and I (KMA), and K and L (BMA). These new conformers tend to have a high ratio of *gauche*-orientation of the alkyl chains.

In particular, KMA showed a different distribution in hexane for which a new conformer was obtained to represent one of the main conformational groupings. This conformation, shown in Figs. 5 and S13 as G, closely resembles an sU-conformation, with a hairpin bend at the cyclopropane group with additional kinks in the chains where the polar head group and mero-chain keto-group are in close proximity.

The principal component plots indicate that most conformations found for single MAs in vacuum or in solution, do not have extended alkyl chains in the trans-orientation, as is suggested by defined WUZ-conformations, but rather constitute a wide range of conformers with bent alkyl chains with high gauche-content. When the alkyl chains of the MAs align closely and in parallel, the trans-orientation is promoted, as seen by the highly ordered W-conformations in which all four alkyl chains are parallel and straight. In MA monolayers and in the cell wall arrangement, the packing of MAs close to each other is likely to promote ordering and trans-orientation of the MA alkyl chains. Therefore, it is not likely that the globular conformations of MAs will be prevalent in these settings. However, at high surface areas in monolayer experiments, where molecules are not tightly packed, conformations with bent and twisted alkyl chains will be more predominant, especially if the molecules are not folded into the W-conformation at these surface areas. Free MAs occurring in mycobacterial biofilms [59], and aqueous environments such as for serodiagnosis, are also likely to occur in more globular conformations.

### FEL of mycolic acids

To further explore key conformations and folding behavior, and provide a rigorous basis for key conformer selection, FELs were used. This method assigns relative energies to conformers based on the frequency that they are represented within a simulation. As such, they reflect a number of the points already extracted from the PCA analysis, namely that there are clear differences in the folding behavior of all MAs, and that they are much more flexible in hexane and show the most defined conformations in water (exemplified by comparing Figs. S16–S27).

A key feature of the FEL is that minima are easily identified and visualized. Clustering can be achieved by applying energy cut-offs to extract conformers that are similar in energy (Fig. S15). This approach affords clearly defined groups corresponding to the most stable structures. Using this approach, the key cluster-averaged minima for AMA, MMA and KMA were extracted for each solvent, using 1, 2 and 3 kcal mol^−1^ cut-offs around the minima on the FEL (Tables S1–S3). The rmsd for the generated cluster structures in water (Tables S4–S6) did not vary appreciably with cut-off (with a couple of exceptions at the 3 kcal mol^−1^ cut-off, where a significant increase was seen), showing that a majority of molecules with similar of conformations can be captured with a cut-off of 1–2 kcal mol^−1^. In each case, the proportion of the structures represented by the cluster groupings was higher than those structures corresponding to WUZ representations. The structures identified indicated a different range of structural variability was important, in line with the preliminary PCA analysis. These differences were most clearly seen in the water simulations, which are also most relevant for the free MAs on which techniques, such as serodiagnosis, presumably rely. Full FELs for each MA under each solvent condition are presented in the Supporting Information (Figs. S16–S27).

**Fig. 6:**
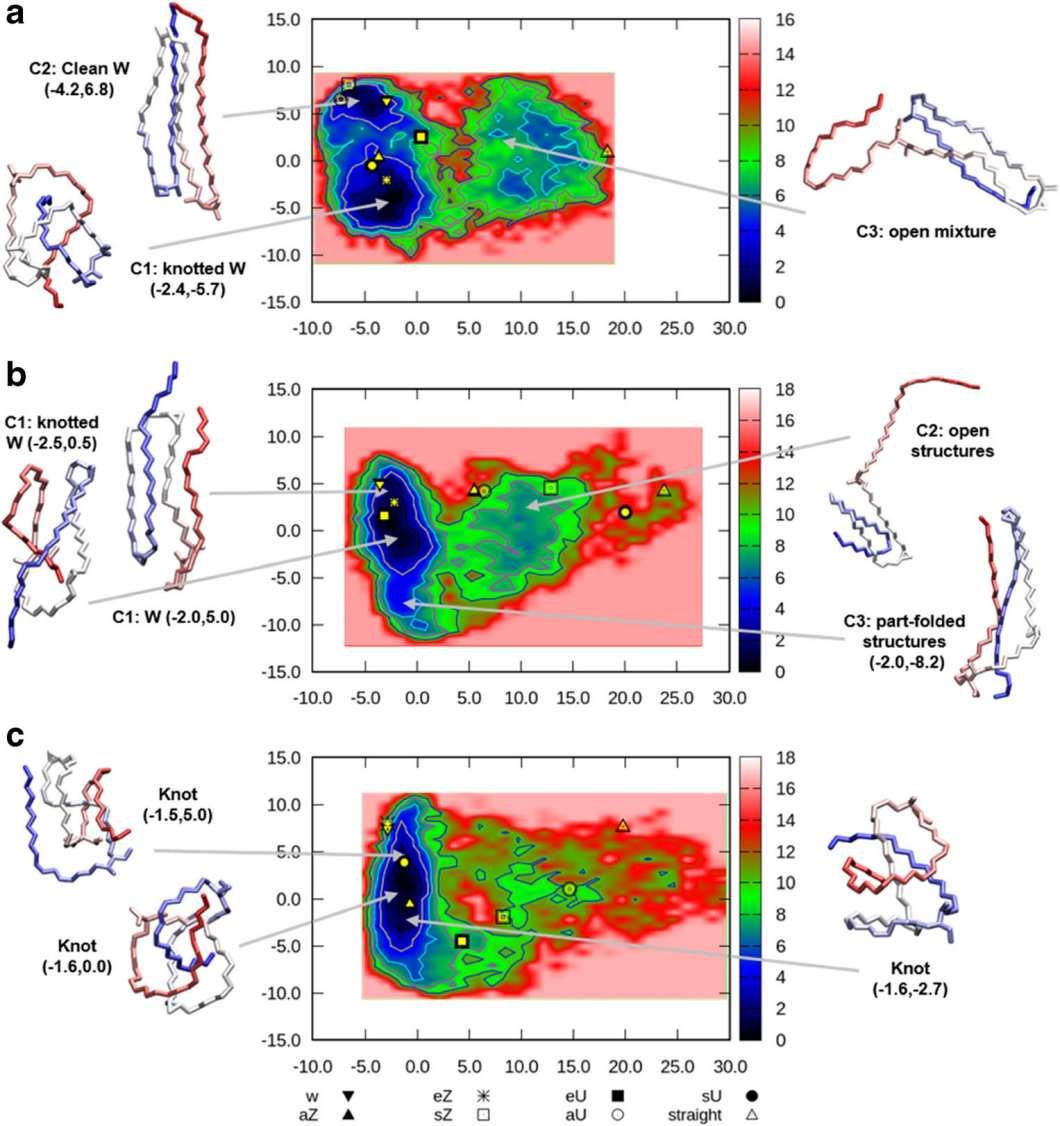
Key structures derived from FELs simulated in water (20 x complete 10 ns simulations) of **a** KMA, **b** AMA and **c** MMA

For KMA, which has the most distinct structuring under the WUZ analysis, three main clusters, a clean W, a knotted W and an open mixture, are identified under the full 10 ns of simulation (Fig. 6a), and only the two ‘W-like’ clusters are in significant proportion in the last 4 ns. Here, these two W-like clusters represent ~19.7 and 36.6% of the simulation frames, respectively, at the 3 kcal mol^−1^ cut-off. In contrast, only 27.7% of structures are classified, across all seven WUZ-conformations for the full 10 ns. The classification of WUZ-defined W structures at 19.2% highlights that FEL analysis enables a more global classification of accessible and closely related conformers with potentially similar structuring and stabilities.

AMA again demonstrates three main clusters initially, collapsing to two key clusters in the last 4 ns (Fig. 6b). A ‘W-like’ cluster has structural representations that are consistent with WUZ-defined W, and other, more knotted, clusters based on the W form. These are not distinguished, as per the KMA case. Under this definition, the W-like structures represent nearly 68.4% of the available frames—an even higher proportion than in KMA. The second lowest-energy cluster represents sZ-related structures (Fig. 6b cluster 3, part-folded structures), contrasting with aZ structures identified by WUZ-analysis as the second major fold for AMA in water. Like KMA, the open structures identified as a cluster in the early part of the simulation collapse by later stages. AMA accounts for about half of the MA content in the *M.* tb cell wall and was shown in previous work to be the most flexible, with the highest percentage of WUZ-conformations. AMA also showed low immune activity and antigenicity experimentally [21, 23]. The flexibility of AMA from WUZ-conformations in the three environments simulated may be more complex, and in fact complicated by a range of knot-like forms that could be difficult to distinguish. The low barrier between different minima may contribute to poor antigenicity. The high percentage of AMA in the cell wall implies that it is key in determining cell wall fluidity and permeability properties, and as such, the impact of cell wall organization will be critical to assess in the future.

MMA shows a particularly interesting profile in water using FEL analysis (Fig. 6c). Here, a single minimum is identified under equilibrated conditions, and, consistent with the low percentage of WUZ structures identified, approximately 70% of the structures are globular-type structures at the 3 kcal mol^−1^ cut-off. MMA occurs naturally with either cis- or trans- cyclopropanation. Experimentally, MMA is the most antigenic, with trans-cyclopropanation showing higher antigenicity than cis-cyclopropanation [21]. Here, the cis-cyclopropane-containing MMA was modelled as it represents the majority as found in *M. tb*. The cis-isomer is extremely immune active, eliciting a distinct inflammatory response, whereas the trans-isomer has largely lost this activity [23]. The knotted structures of the MMA indicated here, and related to those identified for AMA as major contributors, may suggest that the oxygenation constitutes a critical feature of the antigenicity seen for methoxy-species. The role of stereochemistry in folding is likely to reveal further potential mechanisms for immune activity.

### Comparison of WUZ and FEL-based classifications

To see how well the new minima identified by FEL analysis correlated with the WUZ analysis, the structures populating the FEL clusters for water simulations were extracted and correlated with their WUZ classifications for each of the three energy cut-offs (Table S7). Less than half of the structures described by the FEL clusters overlap with WUZ-defined structures, except for cluster 2 of KMA. For this cluster, there was a very high correlation with the W-definition, where just over 80% of this cluster could be defined in this way. This high degree of structuring was supported by the RMSD for this cluster, which was 3.7 and 4.0 Å at the 1 kcal mol^−1^ and 2 kcal mol^−1^ cut-offs, respectively, compared with a value of around 12 for the unclustered portion of the surface. This supports the recognition of KMA as having a propensity to structured folding.

For AMA, cluster 1 correlates with around 11–12% of classic W, with cluster 2 represented by around 40% aZ, whereas the clusters for MMA do not overlap significantly with WUZ-definitions, with the minor cluster 2 (representing <2% of the total structures) being the best defined in this way with between 13.9–18.6% eZ. This comparison of the two mechanisms for defining structures highlights that WUZ conformations, although present, only describe a fraction of conformations for free MAs in vacuum and solvent. FEL-clusters have highlighted that other open, part-folded, and in particular knotted, globular conformations make up a majority of accessible MA conformations, and that these differ depending on the underlying functionality. It may be helpful to apply these latter approaches to cell-wall and membrane-based studies to capture a fuller picture of MA flexibility and conformational scope.

## Conclusions

Various aspects are involved in steering and modifying MA folding. As we have shown before, the unique conformational preferences of each MA show a dependence on the underlying chemical structure [50]. In particular, the functional groups in the MA facilitate folding. This role of functionality in producing defined folds is evidenced by the lack of WUZ-conformations, and distinct FEL minima, identified for the BMA molecule lacking mero-chain functional groups, and the relative flexibility of the backbone reflected in the PCA and FEL analyses for this molecule.

Consistent with monolayer experiments [41, 42, 45–47] and previous simulation results [42, 45, 50], KMA was found to have a preference for more folded conformations as compared to AMA and MMA. The conformational rigidity of KMA, as shown by its folded conformations even at high lateral pressures in monolayers, is expected to alter the cell wall properties, as it does not show the flexibility and fluidity of AMA. The presence of KMA is key in intracellular survival [15]. KMA is also essential for mycobacterial pellicle formation, as well as conferring drug tolerance to the mycobacterium [18]. Only the trans-cyclopropane KMA was modelled here, which shows higher antigenicity than the cis-isomer [21]. The trans-isomer showed an anti-inflammatory response experimentally, compared to the cis-isomer, which elicits a strong inflammatory response [23]. KMA is the only MA modelled here with a trans-cyclopropyl group. KMA shows a high propensity for folding at this group. This trend is consistent with the recent observation that the trans-cyclopropyl group, with its adjacent methyl group, facilitates folding into a hairpin bend, more than cis-cyclopropane [45].

Solvent effects on MA conformations are addressed here for the first time with explicit solvation. FEL-based minima in vacuum and in water were similar for all molecules modelled, suggesting vacuum simulations at much lower cost may provide a good approximation of minimum-energy MA conformations in water. This can be readily rationalized, as a vast majority of the MA composition is the alkyl backbone, so the van der Waals interactions and the specific chain orientations locked by functional group 3D-structure will be the underpinning driving forces for folding in both vacuum and water. In contrast, MA conformations are dispersed among less compactly folded conformations when simulated in hexane for AMA, MMA and BMA. KMA showed a preference for more compactly folded conformations.

Several factors influence the conformations of MAs. Here, the folding at the functional groups from extended chains into defined groups of conformations was demonstrated in vacuum, water and hexane. Utilising the WUZ definitions as primary folding classifications, all MAs afforded W-conformations as the highest percentage, consistent with the W-fold having the lowest energy and thus being the most stable conformer. The lack of folding into WUZ-conformations in a control molecule, BMA, which lacks mero-chain functional groups, shows that the functional groups are crucial in creating folding points in the molecule. FEL analysis indicated that each MA had two to three preferred conformational groupings that could be defined in terms of energetics. Here, only KMA had a majority of structures overlapping with the W-definition, with many structures for AMA and MMA falling outside of the WUZ-definitions in water-based simulations, and this structural demarcation correlates with unique properties for KMA [18, 23].

The unique distribution of conformers obtained for each molecule illustrates that the chemical composition determines the conformational preferences of the MA. In particular, the α-methyl-trans-cyclopropane group of KMA creates a definite folding point, again highlighting that KMA shows an overall preference for more compactly folded conformations, relative to AMA and MMA. This is in agreement with recent results in monolayer experiments and modelling where the trans-cyclopropane group is suggested to facilitate folding more than the cis-cyclopropane group [45].

The role of explicit solvent has been shown here to be important in determining folding, and that longer simulations are necessary to properly model MA folding in water. The explicit solvation of MAs showed that the conformational distributions in vacuum and water simulations were similar enough to define major clusters, although molecules are more flexible to fold and unfold in vacuum. In hexane, mostly open conformations are obtained in a more disperse fashion. KMA is the exception, with a preference for more defined conformations, even in hexane.

Although WUZ-analysis provides us with a method to pinpoint conformations with hairpin bends at the functional groups, it describes only a minority of conformations in solution. PCA analysis and FELs, used for the first time with MA folding in this paper, afford a more complete picture of folding pathways and the distribution of folded states. Based on the FELs, the structures around the minima can be clustered using distinct free energy thresholds. In this way, more structures can be assigned to structurally unique clusters than through the WUZ analysis. New conformations were identified with alkyl chains that are bent and twisted at various points, creating complex patterns of intertwining chains. These more globular conformations of MAs are in the majority for single molecules and may be of relevance to free MAs occurring in biofilms and in experimental applications such as serodiagnosis.

## Electronic supplementary material

ESM 1 Electronic Supplementary Information (ESI) available, including QM fits relating to the parameterization, analyses to confirm equilibration, full PCA maps, full Free Energy Landscapes and WUZ comparison to FEL clustering (PDF). (DOCX 10023 kb)

## Abbreviations

AMA: alpha-mycolic acid
MMA: methoxy-mycolic acid
KMA: keto- mycolic acid
MD: molecular dynamics
FEL: free energy landscape
TB: Tuberculosis
