# Supplementary material for: Revealing solvent-dependent folding behavior of mycolic acids from Mycobacterium tuberculosis by advanced simulation analysis
Source: J Mol Model. 2019 Feb 14;25(3):68. doi: 10.1007/s00894-019-3943-5 (PMC7019640; doi:10.1007/s00894-019-3943-5)
Supplement: Supplementary file 1 — Electronic Supplementary Information (ESI) available, including QM fits relating to the parameterization, analyses to confirm equilibration, full PCA maps, full Free Energy Landscapes and WUZ comparison to FEL clustering (PDF). (DOCX 10023 kb) [file 894_2019_3943_MOESM1_ESM.docx]

## Supporting Information

Revealing solvent-dependent folding behaviour of Mycolic Acids from *Mycobacterium tuberculosis* by advanced simulation analysis

*W. Groenewald, R. Parra Cruz, C. M. Jäger, and A. K. Croft*

**Contents**

Optimised potentials for cyclopropane bond angles (Figs. S1-S6) 2

Energy plots for equilibrated simulations (Figs. S7-S8) 5

Temperature and Pressure plots for equilibrated simulations (Figs. S9-S10) 6

Radius of gyration plots for equilibrated simulations (Fig. S11) 7

Principal component plots for all mycolic acids (Fig. S12-S14) 8

Clustering approach and cutoffs for Free Energy Landscapes (S15) 10

Free Energy Landscapes for all MAs under each solvent condition (Figs. S16-S27) 11

FEL Cluster percentages for water simulations – full vs ‘equilibrated’

Simulations (Tables S1-S3) 15

FEL Cluster all-atom rmsd data - full water simulations (Tables S4-S6) 16

FEL cluster analysis *vs* WUZ classifications - full water simulations (Table S7) 17

**Optimised potentials for cyclopropane bond angles**

**Supplementary figure S1:** Optimised potential for the CT-CT-CY angle using a hybrid ensemble, with OPLS- (with unoptimised cyclopropane parameters) and OPLS+ after fitting to the B3LYP/6-31G* QM energies and forces.

**Supplementary figure S2**: Optimised potential for the CT-CT-CT-CY dihedral angle using a hybrid ensemble with OPLS- (with unoptimised cyclopropane parameters) and OPLS+ after fitting to the B3LYP/6-31G* QM energies and forces.

**Supplementary figure S3**: Optimised potential for the CY-CT-CT-HC dihedral angle using a hybrid ensemble with OPLS- (with unoptimised cyclopropane parameters) and OPLS+ after fitting to the B3LYP/6-31G* QM energies and forces.

**Supplementary figure S4**: Optimised potential for the CT-CT-CY-CY dihedral angle using a hybrid ensemble with OPLS- (with unoptimised cyclopropane parameters) and OPLS+ after fitting to the B3LYP/6-31G* QM energies and forces.

**Supplementary figure S5**: Optimised potential for the CT-CT-CY-HC dihedral angle using a hybrid ensemble with OPLS- (with unoptimised cyclopropane parameters) and OPLS+ after fitting to the B3LYP/6‑31G* QM energies and forces.

**Supplementary figure S6**: Optimised potential for the HC-CT-CY-HC dihedral angle using a hybrid ensemble with OPLS- (with unoptimised cyclopropane parameters) and OPLS+ after fitting to the B3LYP/6‑31G* QM energies and forces.

**Energy plots for equilibrated simulations**

**Supplementary figure S7**: Total energy, potential energy and kinetic energy plots for the 5 ns NVT equilibration of the hexane solvent box.

#
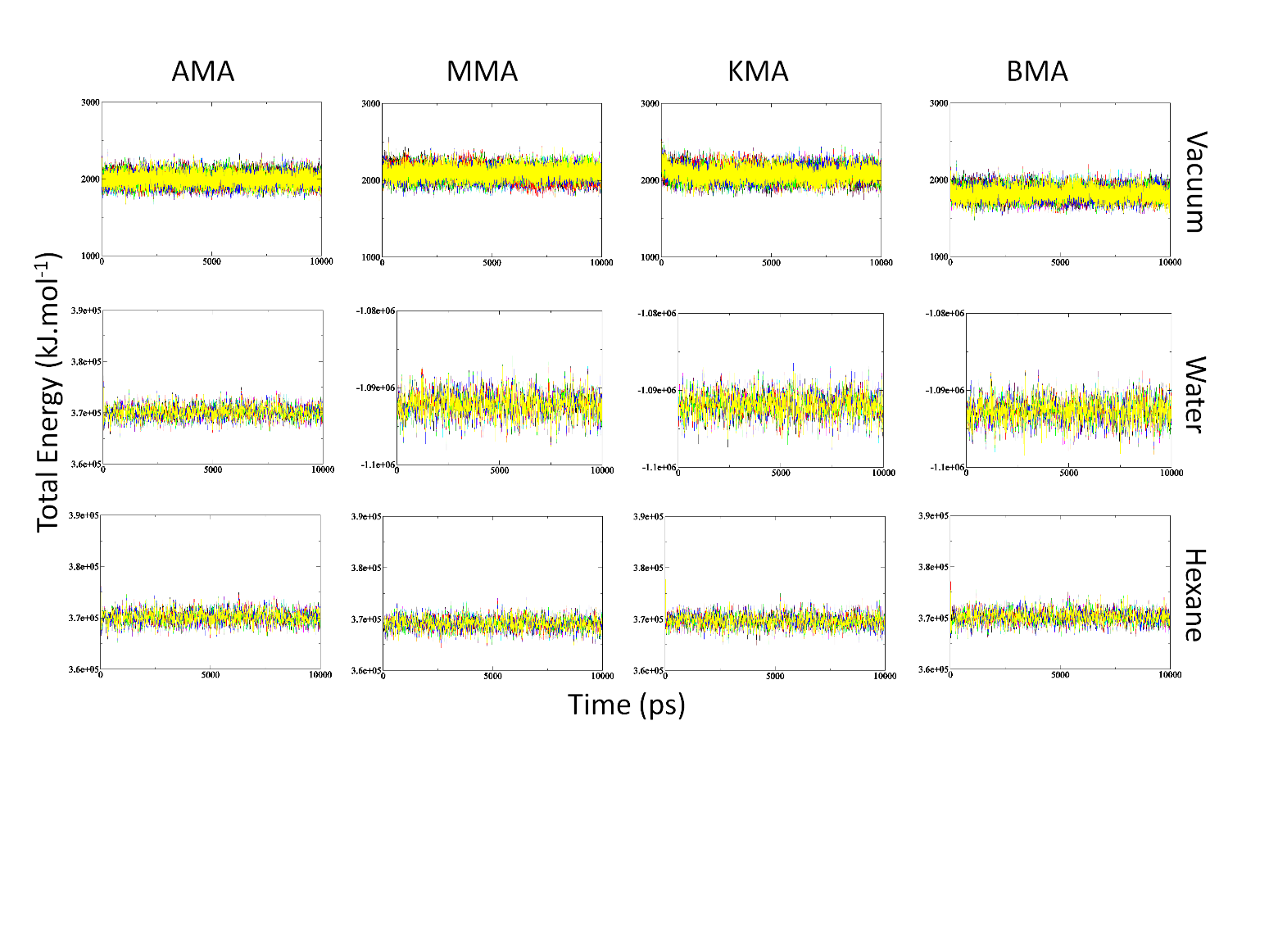


**Supplementary figure S8**: Total energy plots for all molecules studied in vacuum, water and hexane.

**Temperature and Pressure plots for equilibrated simulations**


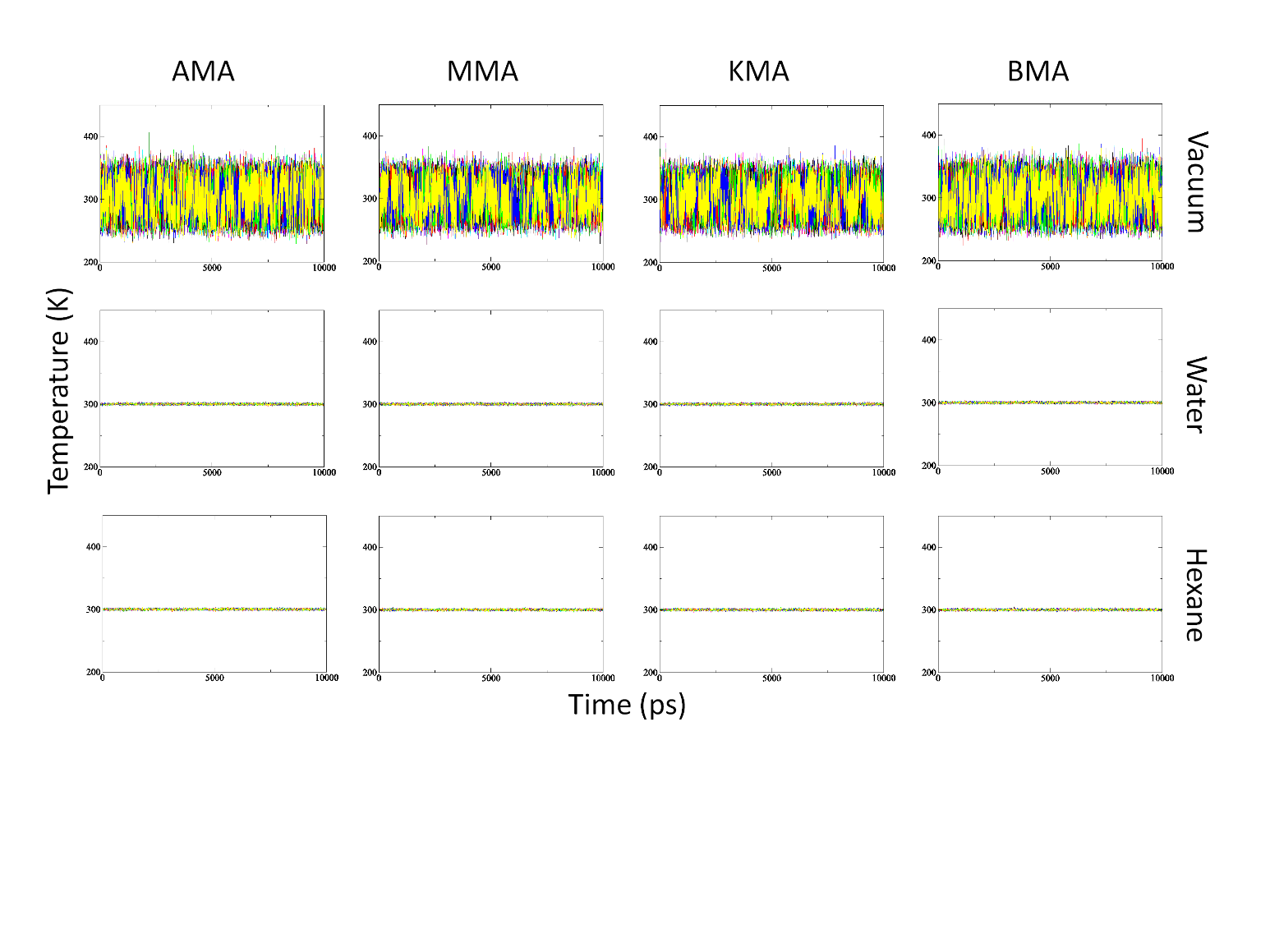


**Supplementary figure S9**: Temperature plots for all molecules studied in vacuum, water and hexane.


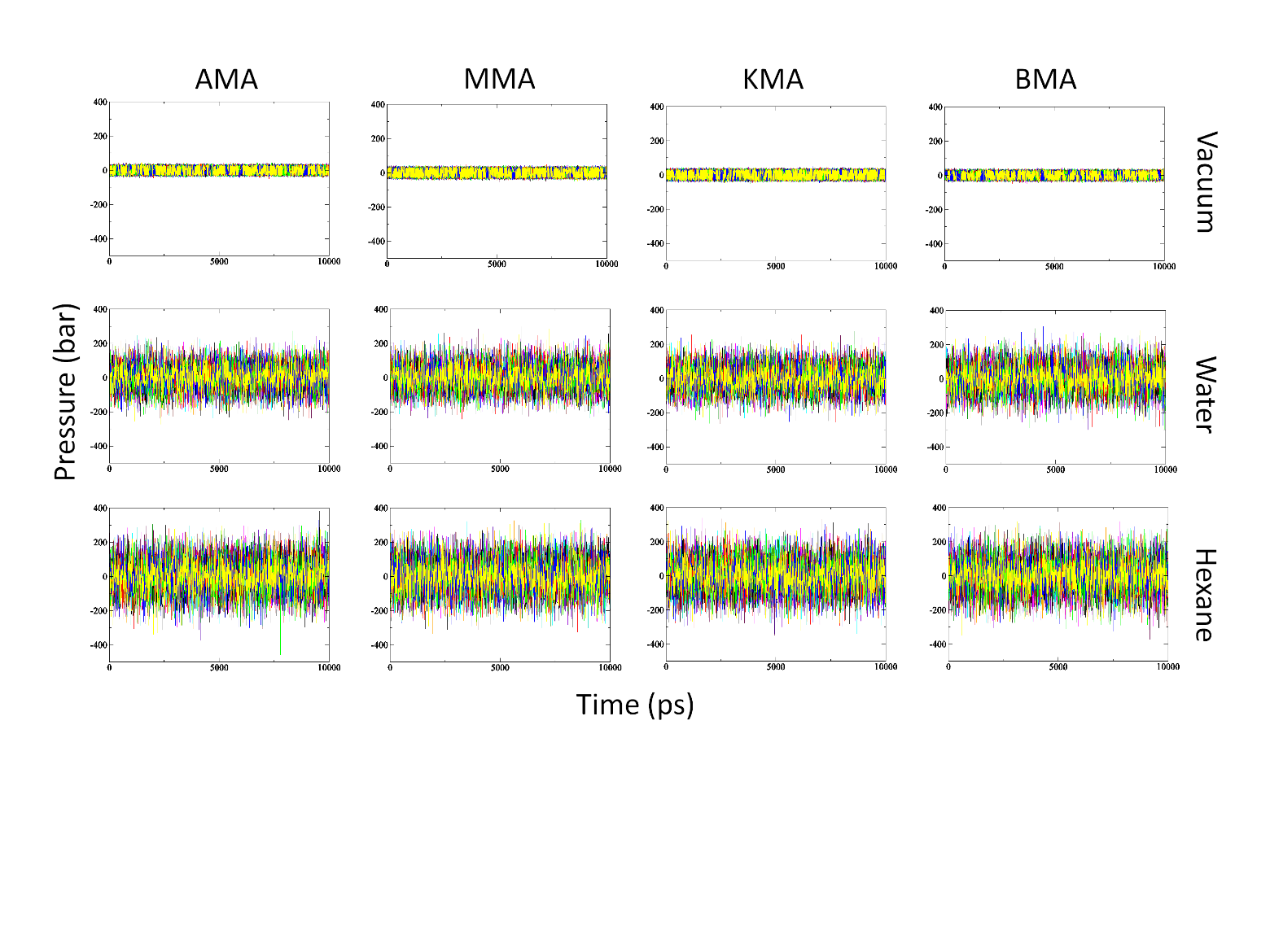


**Supplementary figure S10**: Pressure plots for all molecules studied in vacuum, water and hexane.

**Radius of gyration plots for equilibrated simulations**


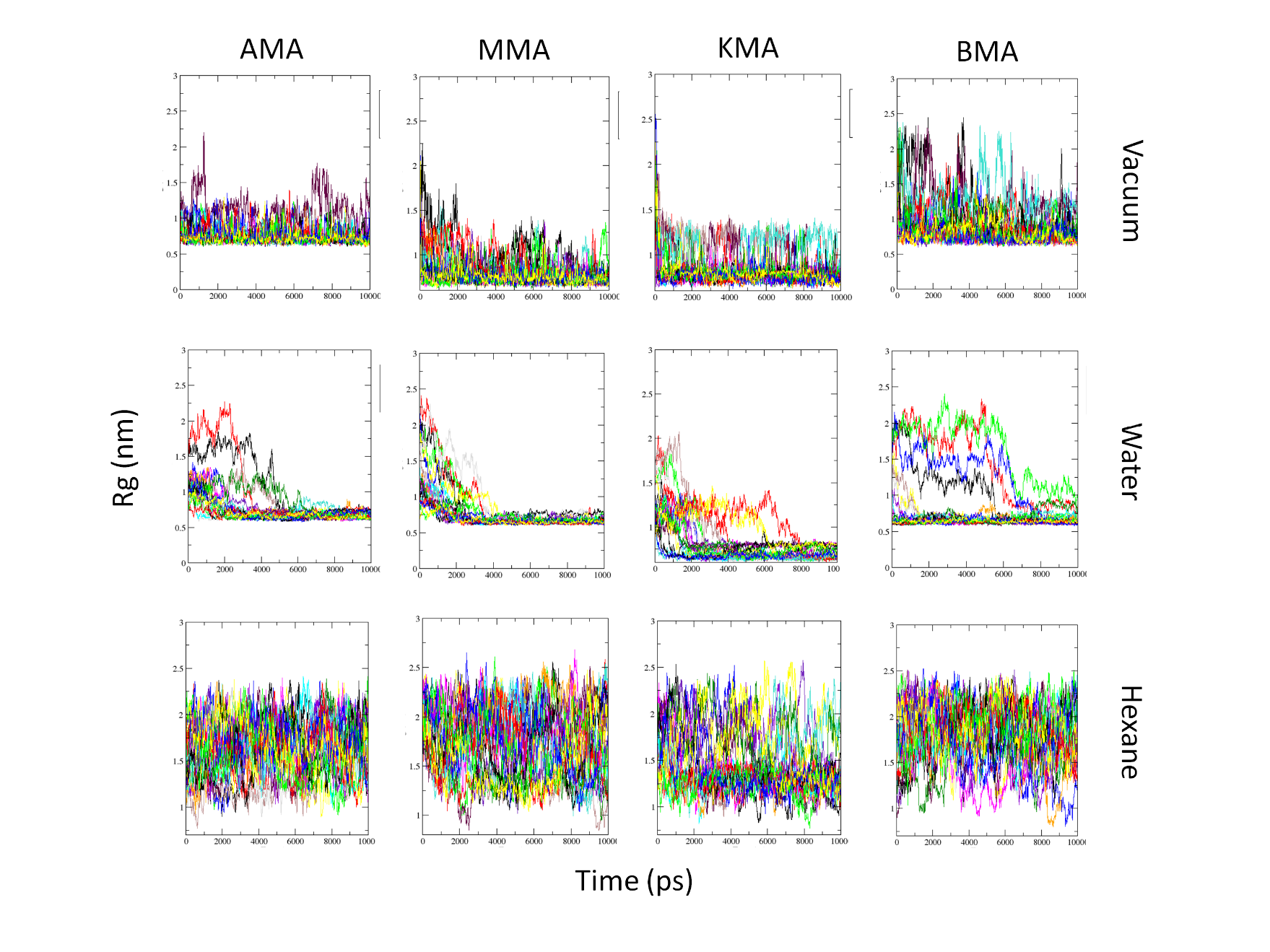


**Supplementary figure S11**: Radius of gyration plots for all molecules studied in vacuum, water and hexane.

**Principal component plots for all mycolic acids**


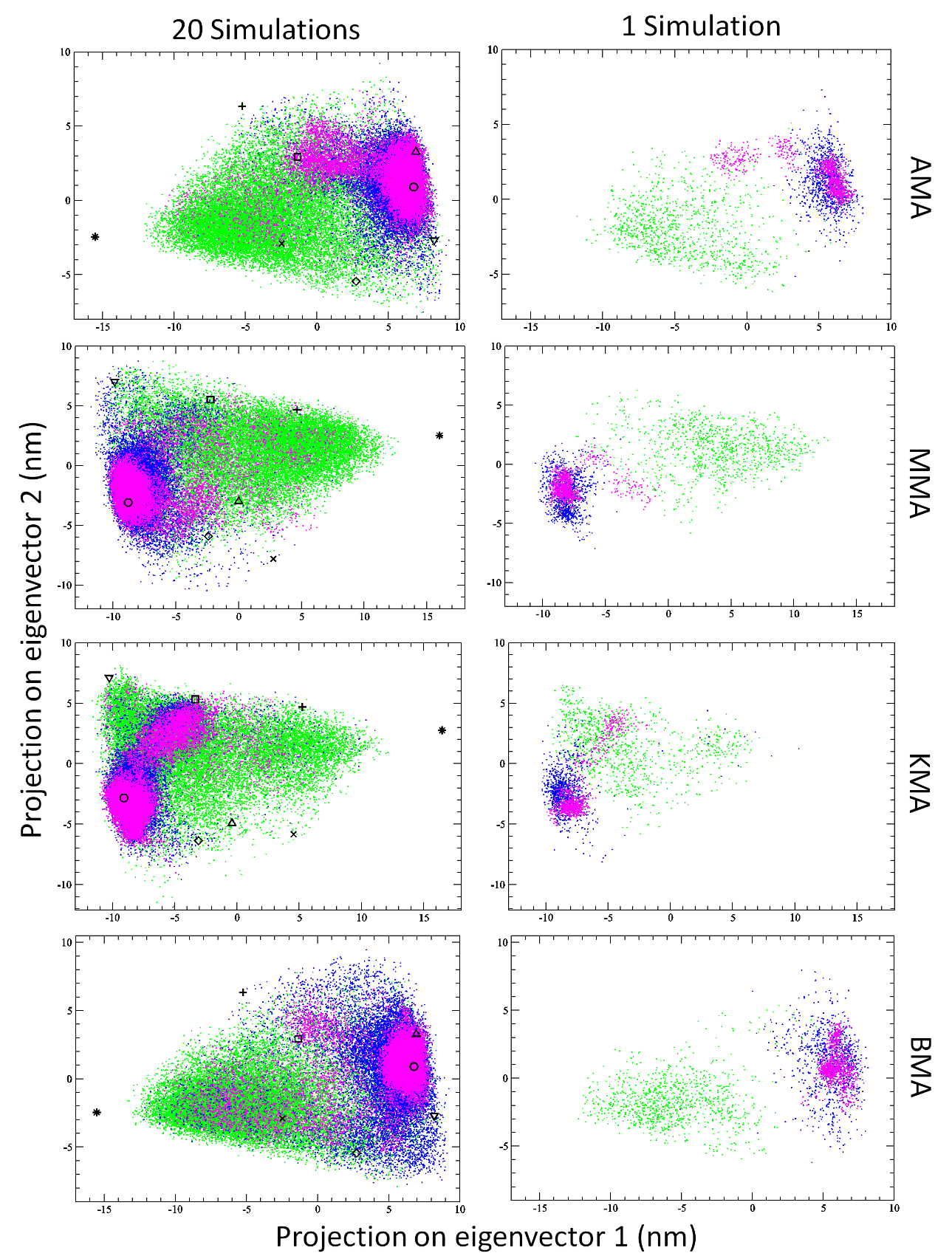

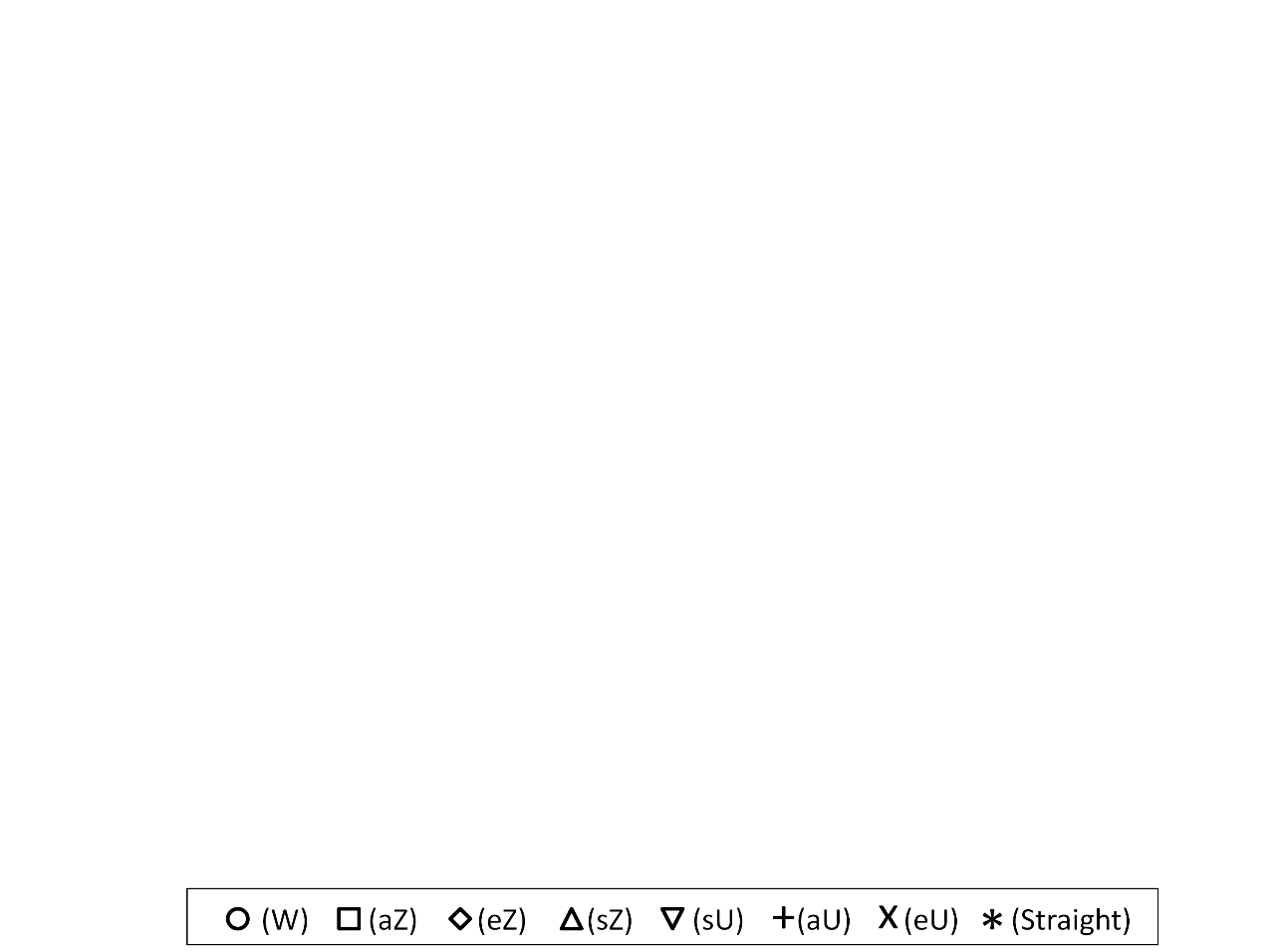


**Supplementary figure S12**: Principal component plots for all molecules modelled in vacuum (blue), water (magenta) and hexane (green). All frames (10 ns) of the twenty replicate simulations (left) and frames for a single example simulation from each solvent (right) are shown.


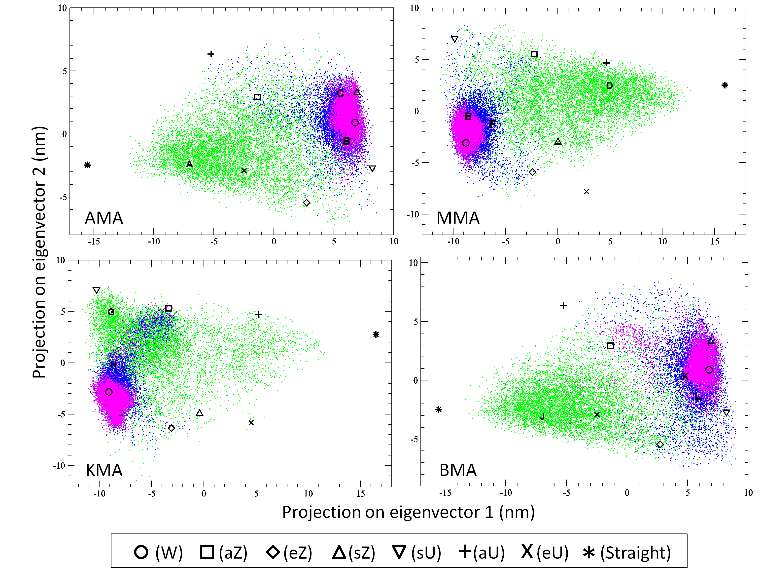


**Supplementary figure S13:** Principal component plots for all molecules modelled in vacuum (blue), water (magenta) and hexane (green). Frames for the last 4 ns of each simulation are shown. The position for the average structures are indicated on the plot for W (circle, ο), aZ (square, 🞎), eZ (diamond, ◊), sZ (triangle, Δ), eU (cross, x) sU (upside-down triangle, ∇) and aU (plus, +). In addition, a completely straight extended conformation with carbon backbone dihedrals of 180**°** is represented by an asterisk (*).


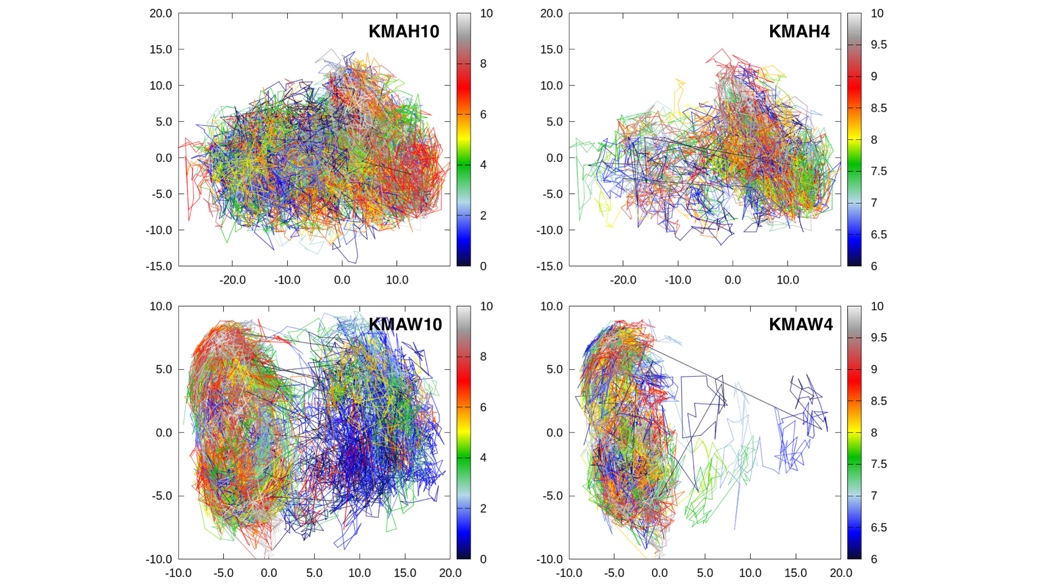


**Supplementary figure 14**. Example of PCA vs time for KMA simulations in hexane (top) and water (bottom): full 10 ns trajectories (left) and last 4 ns of each trajectory (right) showing the reduction in sampled conformational space once the water system is equilibrated. The colour scale represents the simulation time of each individual simulation in nanoseconds.

**Clustering approach and cutoffs for Free Energy Landscapes**


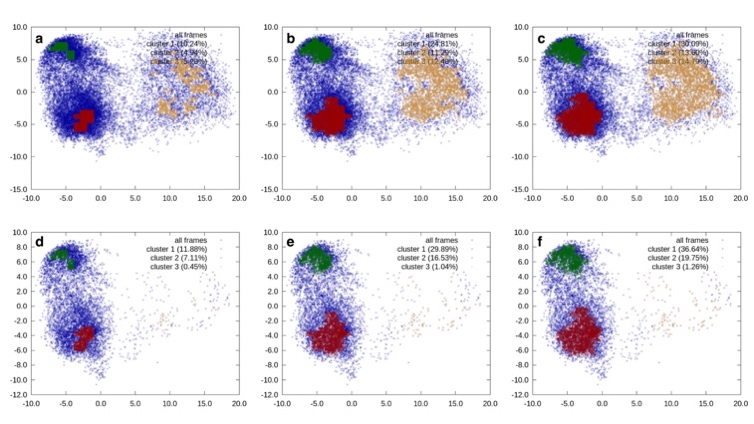


**Supplementary figure S15**. Clustering approach for FEL minima, exemplified for KMA in water. Top: Full trajectory analysis with increasing energetic cutoffs from the minima: a: 1, b: 2 and c: 3 kcal mol^‑1^. Bottom: Analysis of the last 4 ns of simulation indicating more equilibrated structures. Cluster 3 is in low proportion as this constitutes a more open set of structures that collapse to more folded structures during the simulation.

**Free Energy Landscapes for all MAs studied under each solvent condition**


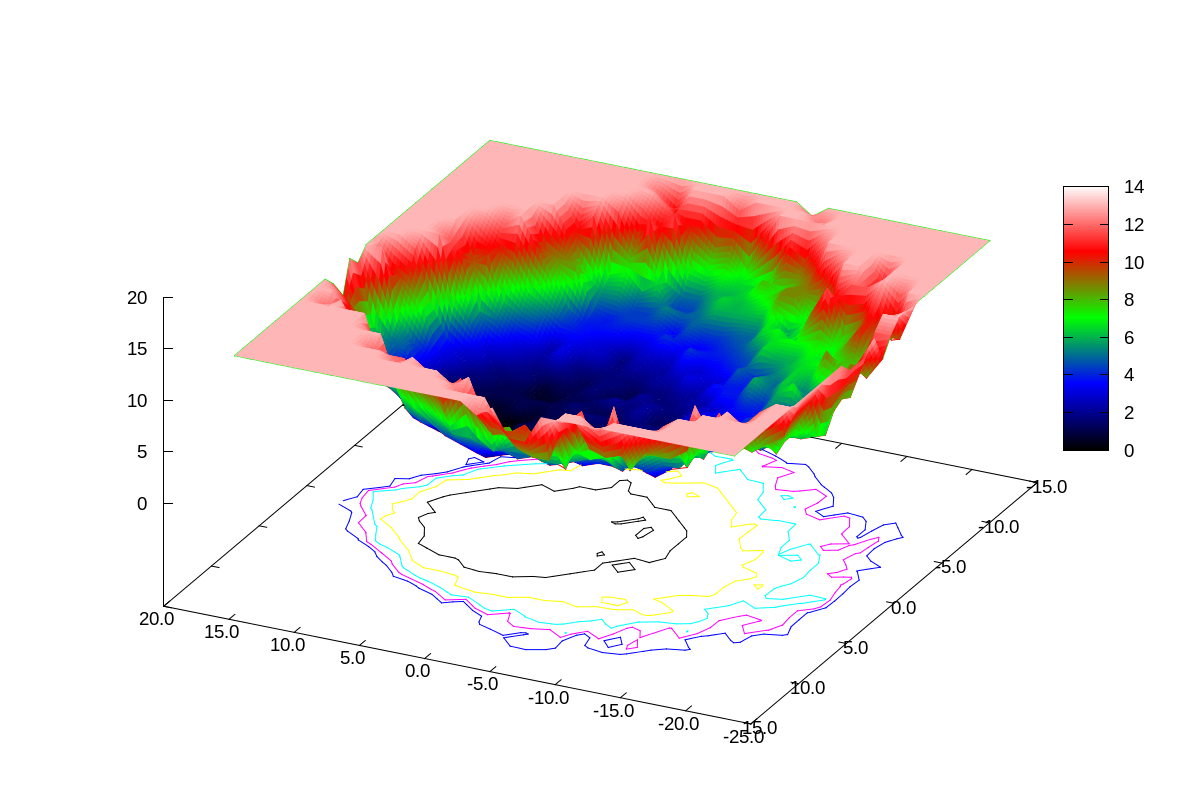


**Supplementary figure S16.** AMA in hexane; Surface generated from 20 10 ns simulations.


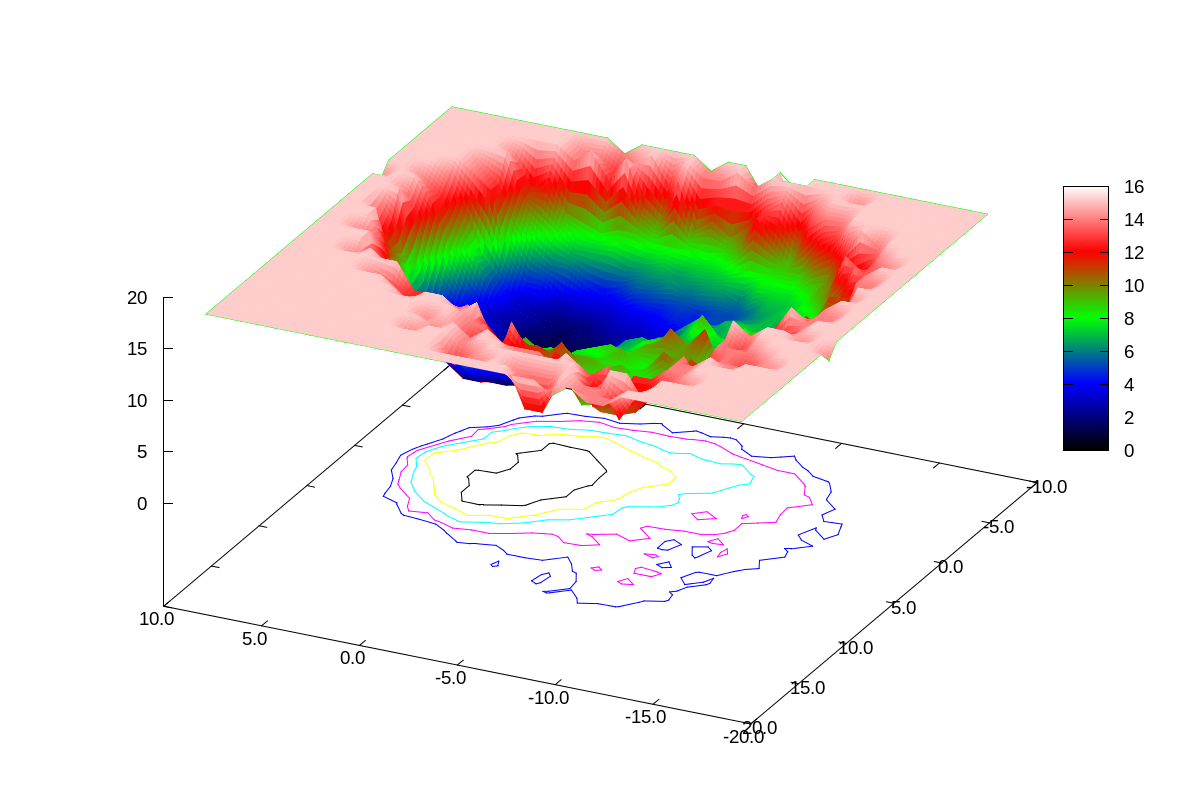


**Supplementary figure S17**. AMA in vacuum; Surface generated from 20 10 ns simulations.


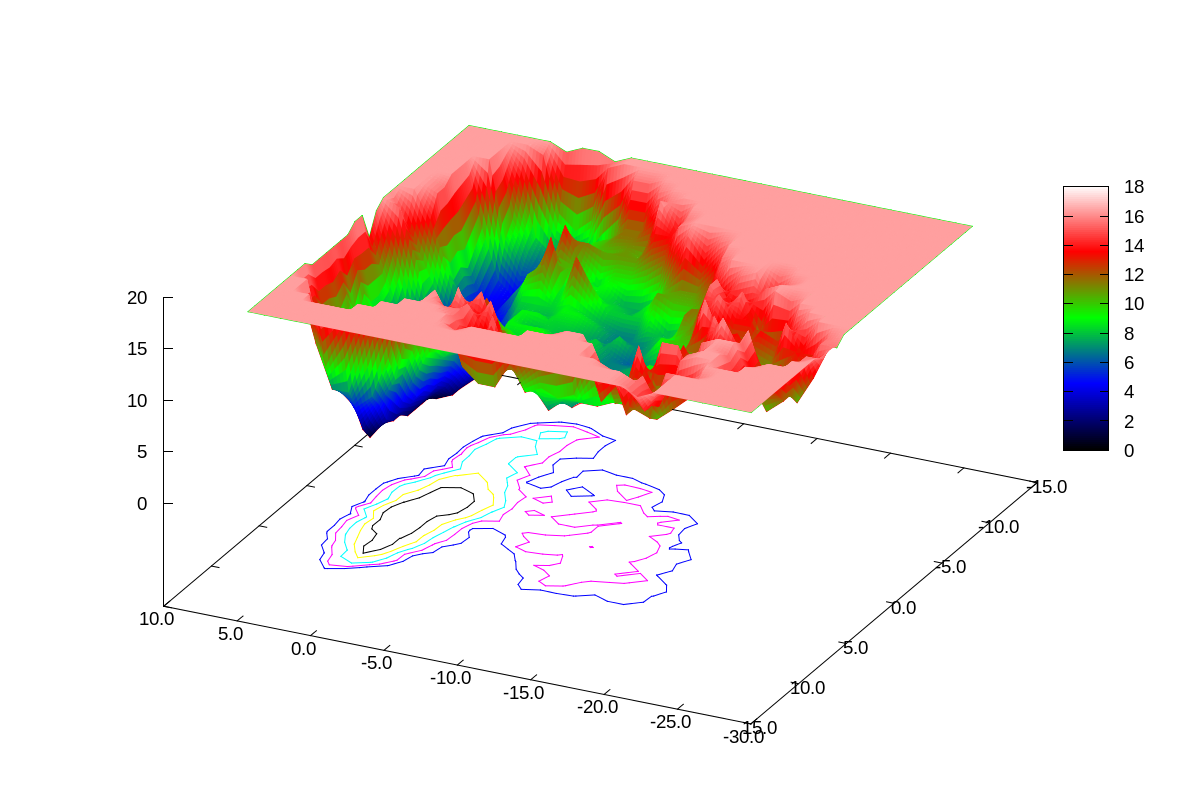


**Supplementary figure S18**. AMA in water; Surface generated from 20 10 ns simulations.


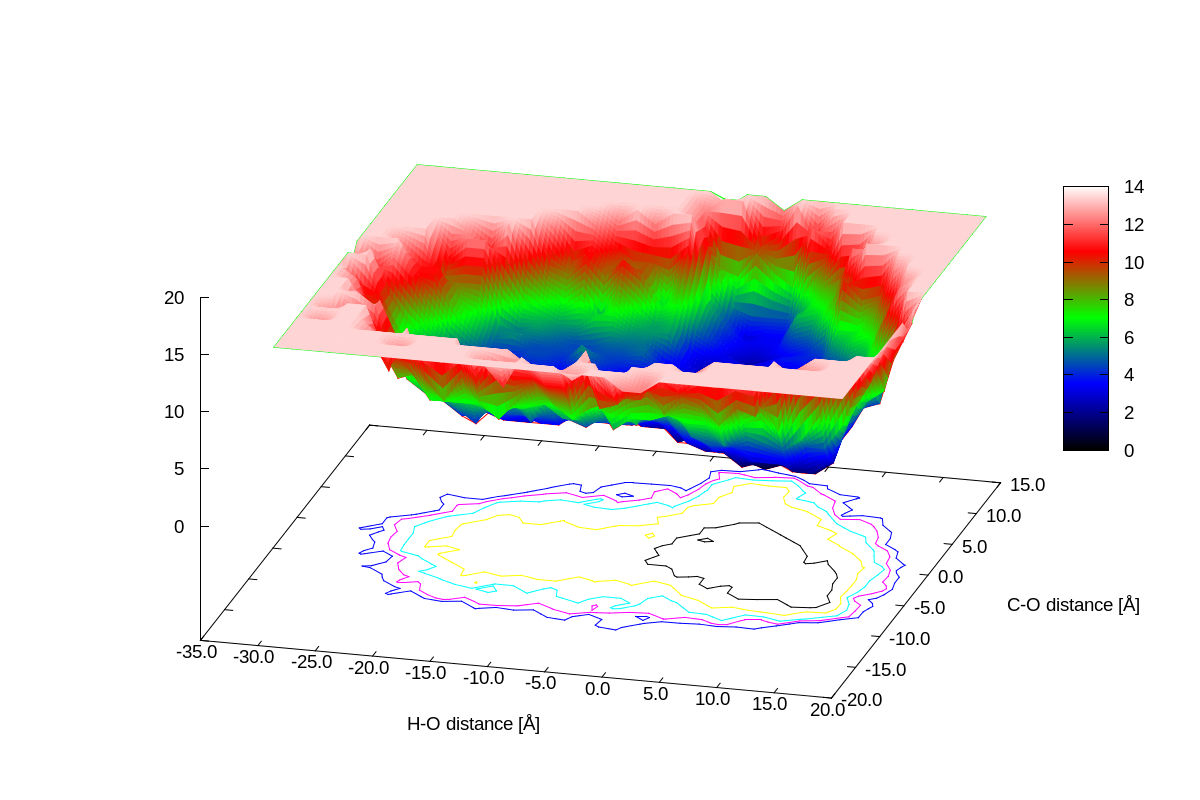
 **Supplementary figure S19**. KMA in hexane; Surface generated from 20 10 ns simulations.


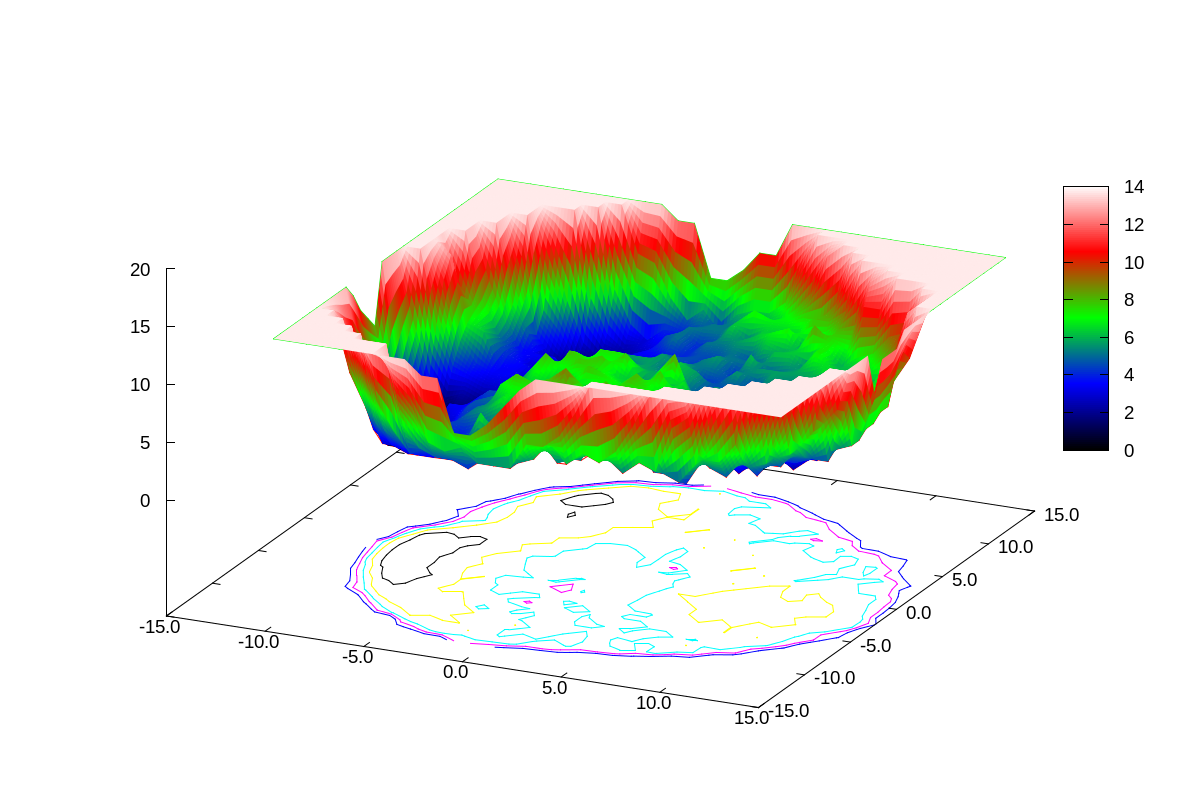


**Supplementary figure S20**. KMA in vacuum; Surface generated from 20 10 ns simulations.


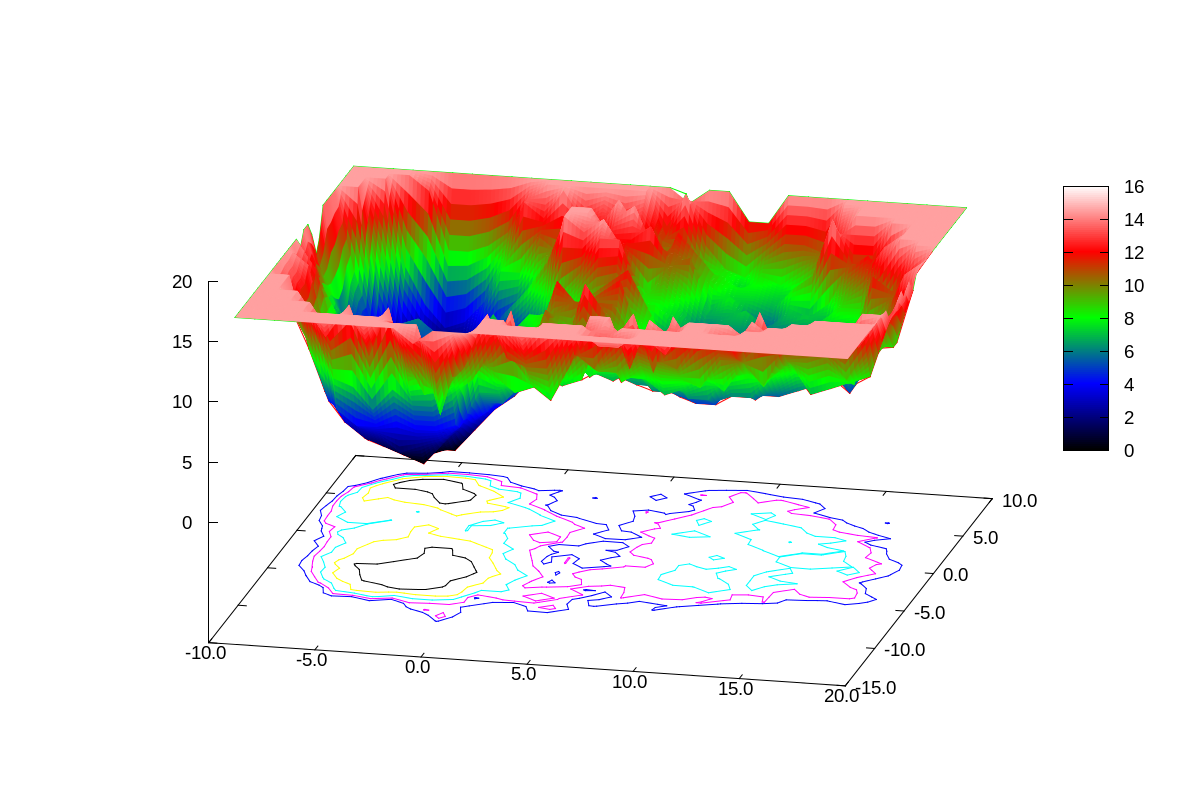


**Supplementary figure S21**. KMA in water; Surface generated from 20 10 ns simulations.


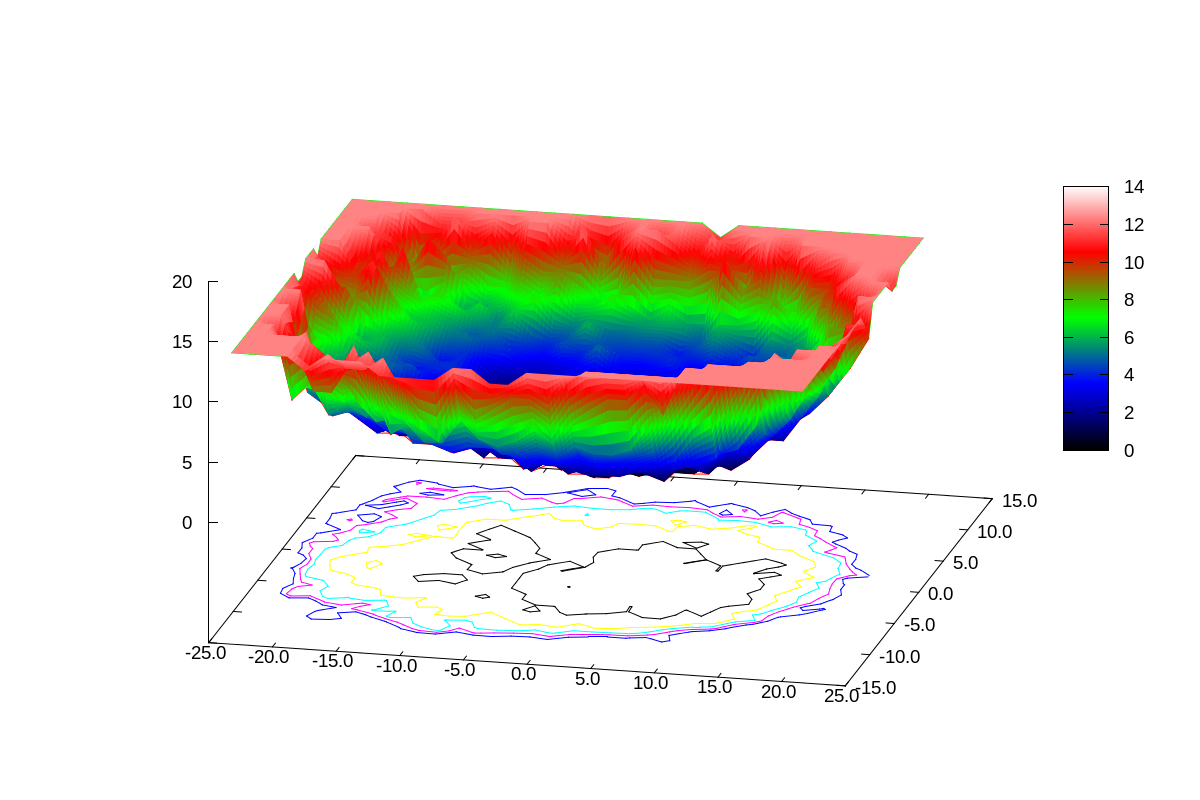


**Supplementary figure S22**. MMA in hexane; Surface generated from 20 10 ns simulations.


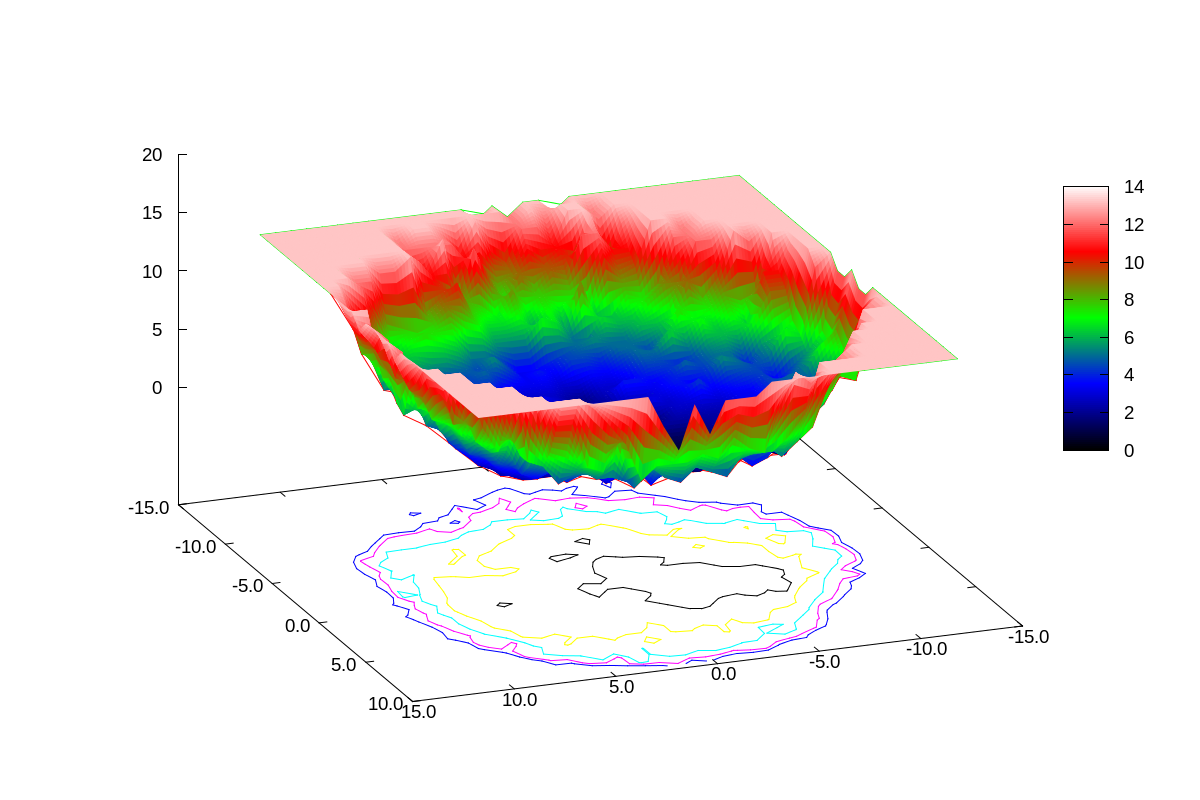


**Supplementary figure S23**. MMA in vacuum; Surface generated from 20 10 ns simulations.


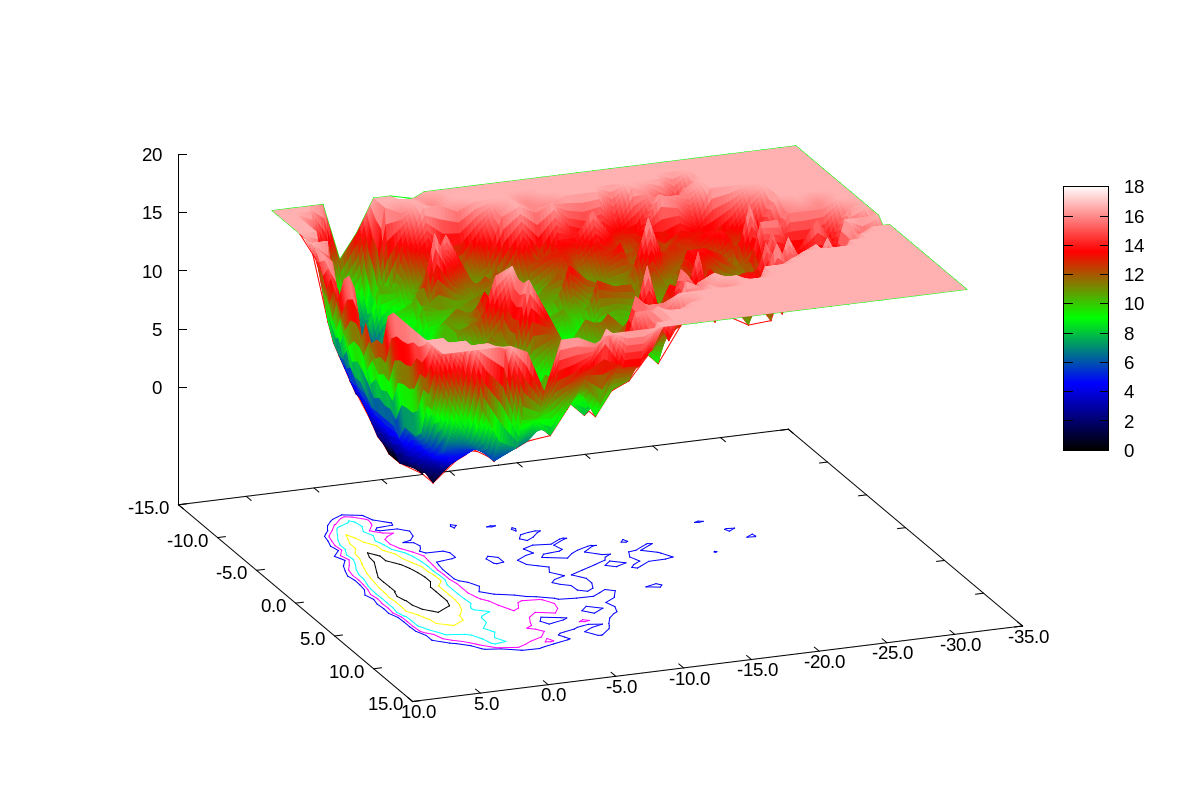


**Supplementary figure S24**. MMA in water; Surface generated from 20 10 ns simulations.


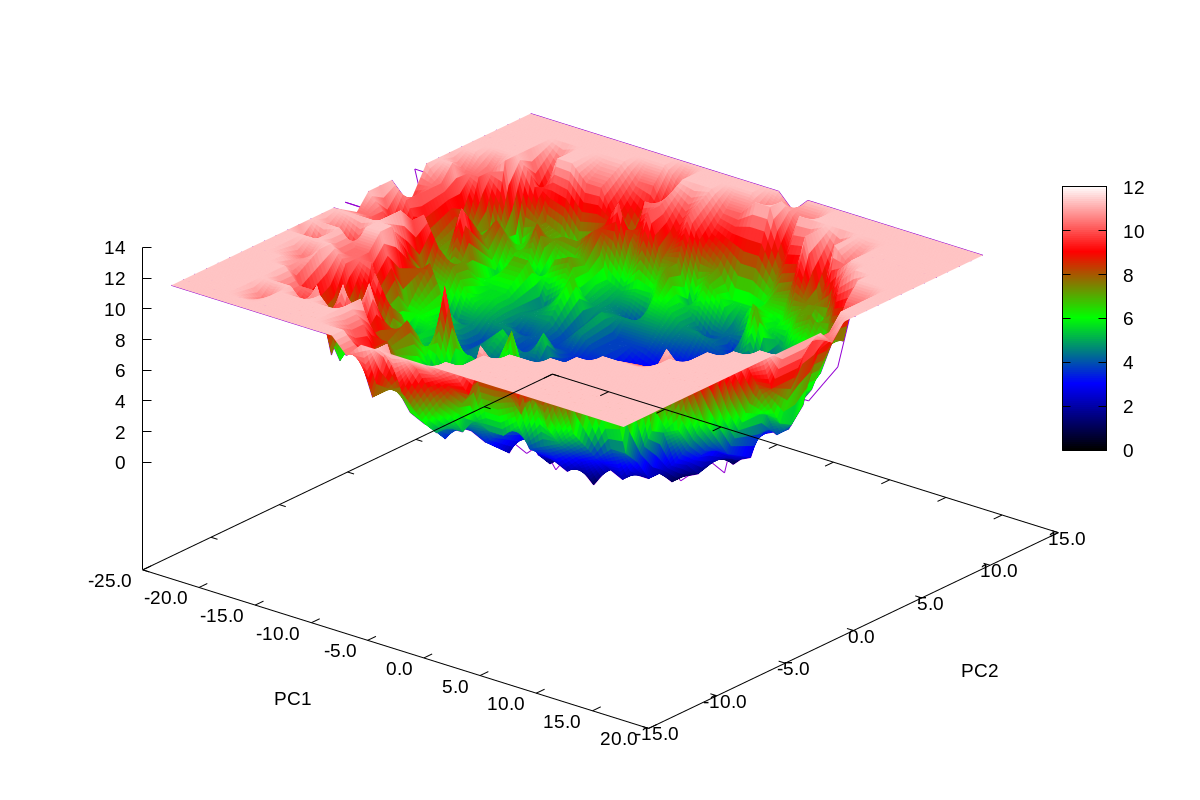


**Supplementary figure S25**. BBA in hexane; Surface generated from 20 10 ns simulations.


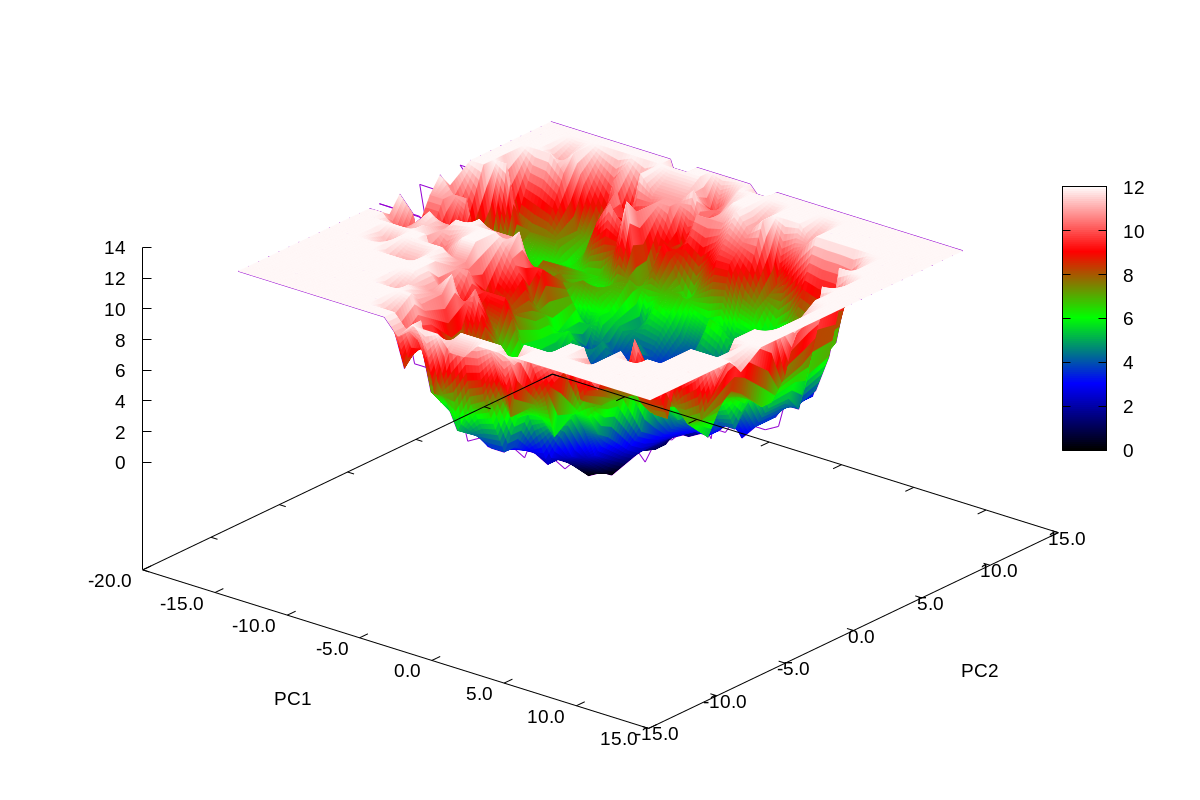


**Supplementary figure S26**. BBA in vacuum; Surface generated from 20 10 ns simulations.


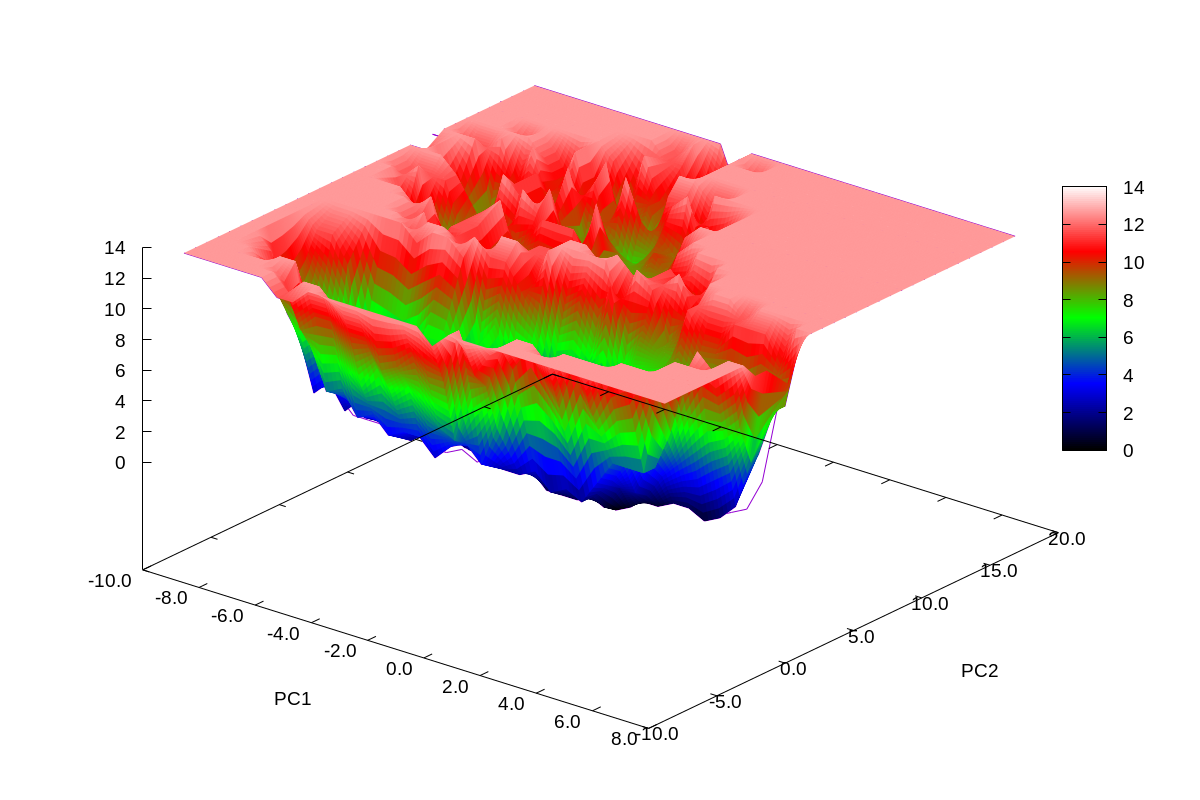


**Supplementary figure S27**. BBA in water; Surface generated from 20 10 ns simulations.

**FEL Cluster percentages for full water simulations (20 x 10 ns) vs ‘equilibrated’ simulations (20 x last 4 ns)**

**Table S1**. KMA water simulation cluster percentages.

|  |  |  | | |  | | |
| --- | --- | --- | --- | --- | --- | --- | --- |
|  | Relative minimum energy [kcal mol^-1^] | Cut-off (10 ns) [kcal mol^-1^] | | | Cut-off (4 ns) [kcal mol^-1^] | | |
|  |  | 1 | 2 | 3 | 1 | 2 | 3 |
| C1 | 0.00 | 10.24 | 24.81 | 30.09 | 11.88 | 29.89 | 36.64 |
| C2 | 0.60 | 4.94 | 11.29 | 13.60 | 7.11 | 16.53 | 19.75 |
| C3 | 4.84 | 5.28 | 12.48 | 14.79 | 0.45 | 1.04 | 1.26 |

**Table S2**. AMA water simulation cluster percentages.

|  | Relative minimum energy [kcal mol^-1^] | Cut-off (10 ns)  [kcal mol^-1^] | | | Cut-off (4 ns)  [kcal mol^-1^] | | |
| --- | --- | --- | --- | --- | --- | --- | --- |
|  |  | 1 | 2 | 3 | 1 | 2 | 3 |
| C1 | 0.00 | 18.35 | 38.33 | 50.73 | 22.76 | 51.29 | 68.39 |
| C2 | 6.11 | 3.69 | 6.63 | 8.92 | 0.00 | 0.03 | 0.06 |
| C3 | 4.07 | 4.88 | 5.85 | 6.80 | 5.01 | 6.17 | 6.68 |

**Table S3**. MMA water simulation cluster percentages.

|  | Relative minimum energy [kcal mol^-1^] | Cut-off (10 ns)  [kcal mol^-1^] | | | Cut-off (4 ns)  [kcal mol^-1^] | | |
| --- | --- | --- | --- | --- | --- | --- | --- |
|  |  | 1 | 2 | 3 | 1 | 2 | 3 |
| C1 | 0.00 | 22.18 | 33.26 | 54.95 | 29.20 | 40.44 | 70.03 |
| C2 | 6.90 | 1.04 | 1.23 | 2.02 | 0.00 | 0.00 | 0.00 |
| C3 | 8.28 | 1.14 | 1.83 | 3.43 | 0.00 | 0.00 | 0.00 |

**FEL Cluster all-atom rmsd data carried out for full water simulations (20 x 10 ns)**

**Table S4**. KMA water simulation cluster rmsd values.

|  | Relative minimum energy [kcal mol^-1^] | Energy cut-off for cluster  [kcal mol^-1^] | | |
| --- | --- | --- | --- | --- |
|  |  | 1 | 2 | 3 |
| C0 (unclustered structures) |  | 11.56 | 11.66 | 12.19 |
| C1 | 0.00 | 7.30 | 6.74 | 6.75 |
| C2 | 0.60 | 3.69 | 3.94 | 4.82 |
| C3 | 4.84 | 10.25 | 10.90 | 10.94 |

**Table S5**. AMA water simulation cluster rmsd values.

|  | Relative minimum energy [kcal mol^-1^] | Energy cut-off for cluster  [kcal mol^-1^] | | |
| --- | --- | --- | --- | --- |
|  |  | 1 | 2 | 3 |
| C0 (unclustered structures) |  | 12.48 | 12.48 | 12.87 |
| C1 | 0.00 | 6.49 | 6.48 | 6.85 |
| C2 | 6.11 | 10.61 | 9.69 | 9.82 |
| C3 | 4.07 | 6.28 | 5.90 | 5.92 |

**Table S6**. MMA water simulation cluster rmsd values.

|  | Relative minimum energy [kcal mol^-1^] | Energy cut-off for cluster  [kcal mol^-1^] | | |
| --- | --- | --- | --- | --- |
|  |  | 1 | 2 | 3 |
| C0 (unclustered structures) |  | 15.95 | 15.90 | 15.60 |
| C1 | 0.00 | 7.12 | 7.18 | 7.27 |
| C2 | 6.90 | 5.89 | 5.85 | 9.89 |
| C3 | 8.28 | 10.56 | 10.47 | 10.45 |

**FEL cluster analysis *vs* WUZ classifications for full water simulations (20 x 10 ns)**

**Table S7**. Percentage of FEL clusters matching WUZ classifications for water simulations.

|  | Cut-off [kcal mol^-1^] | Cluster # | %tot | **% cluster** | | | | | | |
| --- | --- | --- | --- | --- | --- | --- | --- | --- | --- | --- |
|  |  |  |  | **W** | **aZ** | **eZ** | **sZ** | **eU** | **sU** | **aU** |
| KMA water | 1 | 0 | 79.55 | 18.94 | 6.97 | 0.04 | 0 | 0 | 0 | 0 |
|  |  | 1 | 10.24 | 0.10 | 0 | 0 | 0 | 0 | 0 | 0 |
|  |  | 2 | 4.94 | 83.72 | 0 | 0 | 0 | 0 | 0 | 0 |
|  |  | 3 | 5.28 | 0 | 41.63 | 0 | 0 | 0 | 0 | 0 |
|  | 2 | 0 | 51.42 | 19.39 | 5.89 | 0.06 | 0 | 0 | 0 | 0 |
|  |  | 1 | 24.81 | 0.14 | 0.02 | 0 | 0 | 0 | 0 | 0 |
|  |  | 2 | 11.29 | 81.54 | 0 | 0 | 0 | 0 | 0 | 0 |
|  |  | 3 | 12.48 | 0 | 37.74 | 0 | 0 | 0 | 0 | 0 |
|  | 3 | 0 | 41.51 | 19.61 | 4.78 | 0.07 | 0 | 0 | 0 | 0 |
|  |  | 1 | 30.09 | 0.30 | 0.02 | 0 | 0 | 0 | 0 | 0 |
|  |  | 2 | 13.60 | 80.73 | 0 | 0 | 0 | 0 | 0 | 0 |
|  |  | 3 | 14.79 | 0 | 38.91 | 0 | 0 | 0 | 0 | 0 |
| AMA water | 1 | 0 | 73.09 | 9.49 | 5.35 | 0 | 0 | 0 | 0 | 0 |
|  |  | 1 | 18.35 | 11.05 | 0.11 | 0 | 0 | 0 | 0 | 0 |
|  |  | 2 | 3.69 | 0 | 41.15 | 0 | 0 | 0 | 0 | 0 |
|  |  | 3 | 4.88 | 0.10 | 0 | 0 | 0 | 0 | 0 | 0 |
|  | 2 | 0 | 49.19 | 9.24 | 5.49 | 0 | 0 | 0 | 0 | 0 |
|  |  | 1 | 38.33 | 11.52 | 0.07 | 0 | 0 | 0 | 0 | 0 |
|  |  | 2 | 6.63 | 0 | 41.06 | 0 | 0 | 0 | 0 | 0 |
|  |  | 3 | 5.85 | 0.09 | 0 | 0 | 0 | 0 | 0 | 0 |
|  | 3 | 0 | 33.56 | 7.90 | 5.22 | 0 | 0 | 0 | 0 | 0 |
|  |  | 1 | 50.73 | 12.44 | 0.06 | 0 | 0 | 0 | 0 | 0 |
|  |  | 2 | 8.92 | 0 | 41.1 | 0 | 0 | 0 | 0 | 0 |
|  |  | 3 | 6.80 | 0.07 | 0 | 0 | 0 | 0 | 0 | 0 |
| MMA water | 1 | 0 | 75.64 | 5.65 | 0.11 | 1.66 | 0 | 0 | 0 | 0 |
|  |  | 1 | 22.18 | 0.47 | 0 | 0.02 | 0 | 0 | 0 | 0 |
|  |  | 2 | 1.04 | 0 | 0 | 13.93 | 0 | 0 | 0 | 0 |
|  |  | 3 | 1.14 | 0 | 0 | 2.63 | 0 | 0 | 0 | 0 |
|  | 2 | 0 | 63.96 | 6.52 | 0.13 | 1.87 | 0 | 0 | 0 | 0 |
|  |  | 1 | 33.26 | 0.68 | 0 | 0.02 | 0 | 0 | 0 | 0 |
|  |  | 2 | 1.23 | 0 | 0 | 16.24 | 0 | 0 | 0 | 0 |
|  |  | 3 | 1.83 | 0 | 0 | 2.46 | 0 | 0 | 0 | 0 |
|  | 3 | 0 | 39.58 | 9.15 | 0.18 | 2.46 | 0 | 0 | 0 | 0 |
|  |  | 1 | 54.94 | 1.36 | 0 | 0.01 | 0 | 0 | 0 | 0 |
|  |  | 2 | 2.04 | 0.24 | 0.24 | 18.61 | 0 | 0 | 0 | 0 |
|  |  | 3 | 3.43 | 0 | 0.15 | 2.33 | 0 | 0 | 0 | 0 |
